# Identification of a gene expression driven progression pathway in myxoid liposarcoma

**DOI:** 10.18632/oncotarget.2023

**Published:** 2014-05-27

**Authors:** Loris De Cecco, Tiziana Negri, Silvia Brich, Valentina Mauro, Fabio Bozzi, GianPaolo Dagrada, Vittoria Disciglio, Roberta Sanfilippo, Alessandro Gronchi, Maurizio D'Incalci, Paolo G. Casali, Silvana Canevari, Marco A. Pierotti, Silvana Pilotti

**Affiliations:** ^1^ Functional Genomics and Bioinformatics, Department of Experimental Oncology and Molecular Medicine, Fondazione IRCCS Istituto Nazionale dei Tumori, Milan, Italy; ^2^ Laboratory of Experimental Molecular Pathology, Department of Diagnostic Pathology and Laboratory, Fondazione IRCCS Istituto Nazionale dei Tumori, Milan, Italy; ^3^ Adult Mesenchymal Tumor Medical Oncology Unit, Cancer Medicine Department, Fondazione IRCCS Istituto Nazionale dei Tumori, Milan, Italy; ^4^ Department of Surgery, Fondazione IRCCS Istituto Nazionale dei Tumori, Milan, Italy; ^5^ Department of Oncology, IRCCS, Istituto di Ricerche Farmacologiche Mario Negri, Milan, Italy; ^6^ Scientific Directorate, Fondazione IRCCS Istituto Nazionale dei Tumori, Milan, Italy

**Keywords:** myxoid liposarcoma, progression to round cell, gene expression array, epigenetic deregulation, stemness related genes, fast cell cycle related genes

## Abstract

Aim: to investigate the events involved in the progression of myxoid liposarcoma (MLS).

Gene expression profiling and immunohistochemical/biochemical analyses were applied to specimens representative of the opposite ends of the MLS spectrum: pure myxoid (ML) and pure round cell (RC) liposarcomas.

The analyses revealed the involvement of both coding and non coding RNAs (SNORDs located in DLK1-DIO3 region) and support a model of stepwise progression mainly driven by epigenetic changes involving tumour vascular supply and tumoral cellular component. In this model, a switch in the vascular landscape from a normal to a pro-angiogenic signature and the silencing of DLK1-DIO3 region mark the progression from ML to RC in concert with the acquisition by the latter of the over-expression of YY1/C-MYC/HDAC2, together with over-expression of genes involved in cell proliferation and stemness: *MKNK2, MSX1* and *TRIM71*.

Taken together, these findings strongly suggest that to progress from ML to RC liposarcoma the cells have to overcome the epigenetic silencing restriction point in order to reset their new stem-like differentiation signature. Our findings provide a first attempt at identifying the missing links between ML and RC liposarcomas, that may also have broader applications in other clinico-pathological settings characterised by a spectrum of progression.

## INTRODUCTION

Myxoid liposarcomas (MLS) encompass myxoid (ML) and round cell (RC) variants initially believed to be distinct diseases [1] and currently regarded as the well- and the poorly-differentiated extremes of a single spectrum. Molecularly MLS is characterised by the recurrent *FUS-DDIT3* and rarely (10%) *EWSR1-DDIT3* balanced translocations. Within the spectrum of MLS, the fusion proteins that make the cells “immature” by blocking lipogenic differentiation play a role in tumour initiation, but little is known about the molecular determinants associated with their progression to RCs.

Activation of the AKT pathway sustained by *PIK3CA* [2] and *PTEN* [3,4] mutations, and signalling by growth factor receptors such as RET and IGF1R, have been recently correlated with the increased aggressiveness of RC [3-5]. In the clinical setting, however, the most significant predictor of a poor outcome in patients with MLS remains the amount of round cell (RC) component (>5%) as this increases the risk of metastases [6]. It worth noting that the five-year survival rate among patients with MLS ranges from 20-70%, and is shortest in those with RC [7].

To develop a predictor of outcome in liposarcoma patients, Gobble et al. analyzed microarray-based gene expression profiling of 140 samples [8]. This case material included 17 ML and 12 RC defined as MLS with RC component >5%.

The aim of this study is to elucidate the molecular events involved in RC progression by means of microarray-based gene expression profiling and gene-by-gene hypothesis-driven analysis. Two small series of MLS specimens (the first used for the training and the second for validation) were selected in such a way as to be representative of the two extremes of the MLS spectrum: pure myxoid (about 0% of RC component) and RC specimens (≥80% of RCs) [1].

## RESULTS

### Identification of gene expression profiles differentially expressed in myxoid and round cell liposarcomas

**Figure 1:**
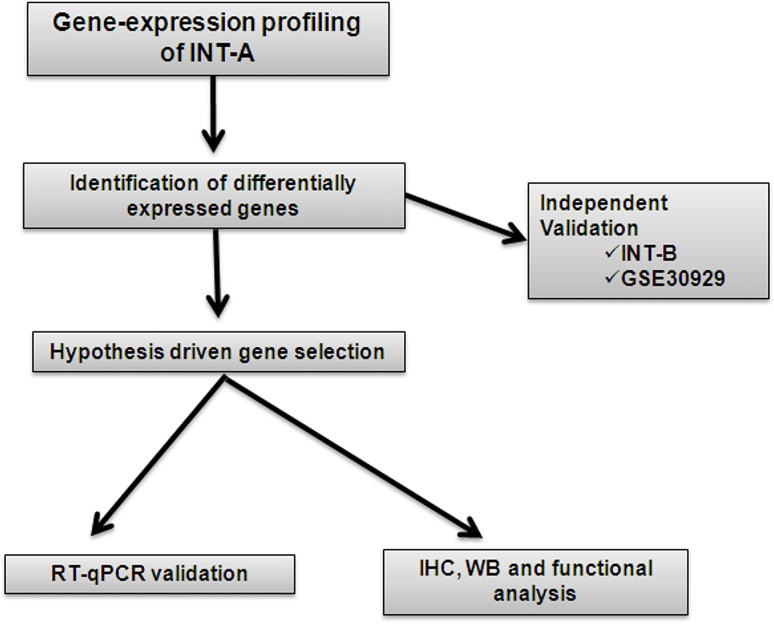
Study outline

**Figure 2:**
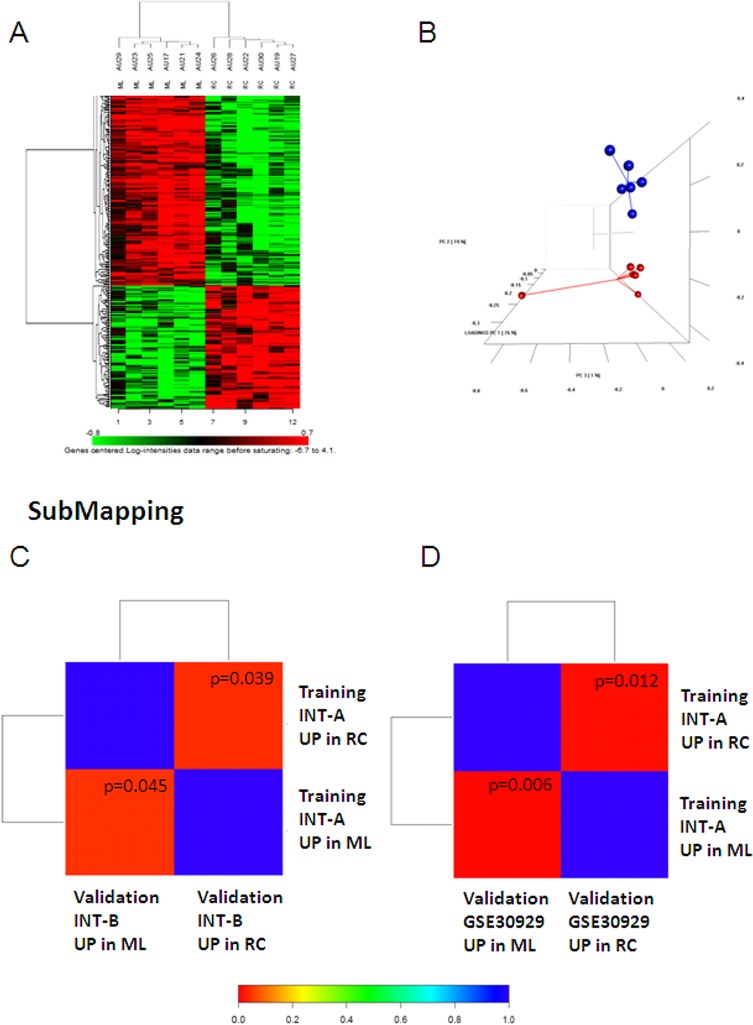
(A and B) Genes differentially expressed in the INT-A dataset. (A) Heatmap of the genes differentially expressed after imposing an FDR of <0.1. (B) The differentially expressed genes visualised by PCA divided the samples into two well-defined groups corresponding to ML (blue) and RC (red). Sub-class mapping (SubMap) analysis comparing the genome-wide molecular pattern identified in INT-A with the patterns identified in the INT-B (C) and GSE30929 data sets (D). Red indicates high confidence in correspondence; blue indicates a lack of correspondence. P values are given in the boxes.

Figure 1 shows the workflow of the study. In order to identify the gene expression pattern modulated in ML and RC liposarcomas, a training set of 12 FFPE samples (6 ML and 6 RC; case material INT-A, see Supplementary Table S1 for the clinical/pathological/molecular characteristics of the patients) was initially selected and profiled using the Illumina whole-genome DASL assay. In this dataset, 16,859 transcripts were detected, and 307 probes, corresponding to 298 unique genes, were identified as differentially expressed by means of class comparison analysis using a false discovery rate (FDR) of <10%: 115 probes up-regulated in RC and 192 up-regulated in ML (Figure 2A). The probability of finding 307 probes significant by chance if there were no real differences between the classes was 0.00649, as determined by the global test. Principal component analysis (PCA) indicated that the samples were distributed in two main clusters matching the ML and RC samples (Figure 2B).

For validation purposes, we assessed the extent to which the molecular patterns differentially expressed in the training set were similar to those in a new cohort of 12 frozen samples (6 ML and 6 RC; case material INT-B, see Supplementary Table S2), and a public dataset (GSE30929) [8] containing 17 ML and 12 RC liposarcomas. Both data sets were first analysed separately in order to define the genes differentially expressed in ML and RC liposarcomas. By imposing a FDR of <10%, we identified 64 genes in INT-B and 58 in GSE30929 (Supplementary Figure S1). Using a bioinformatic method that assesses the correspondence of molecular patterns in different datasets (subclass mapping), we found significant regulation of the same genes (Figures 2C and 2D), thus providing evidence of the reliability of the genes differentially expressed in the training set.

### Selection of genes for protein analysis

A list of the 174 modulated genes in the ML and RC variants was generated by applying a filter based on a |log2 (fold change)|>2 and a permutation p-value <0.001 to the INT-A data set (Supplementary Table S5). On the basis of published data and given the biological characteristics of MLS, we focussed on the most differentially expressed genes in three main categories: i) those involved in angiogenesis; ii) those located in the DLK1-DIO3 region on chromosome 14q32; and iii) those involved in cell proliferation and staminal status. These were validated in the INT-A and INT-B series by means of qRT-PCR and the results are shown in Table 1. The differential expression was tested at protein level by IHC and/or WB for all markers, but TRIM71 and non coding RNAs (SNORDs) of DLK1-DIO3 region.

**Table 1:** Evaluation of differentially expressed genes /miRNAs involved in the proposed model of ML to RC progression, at RNA and protein level

Gene/miRNA	microarrays Fold change (RC/ML m)		RTq-PCR Training set (RC/ML)		RTq-PCR Validation set (RC/ML)		Protein evaluation
	Training set	Validation set	Geometric mean of ratios	Parametric p-value	Geometric mean of ratios	Parametric p-value	
Angiogenesis							
EGFL6	0.068	0.17	0.042	0.0010987	0.0086	0.0010582	IHC
EGFL7	0.21	0.89	0.07	0.0005039	0.44	0.0463813	IHC
hsa-miR-6086# (EGFL6)	ND	ND	0.22	0.0146451	ND	ND	NA
hsa-miR-126# (EGFL7)	ND	ND	0.13	0.012225	ND	ND	NA
GREM2	5.37	2.94	8.88	0.0085956	6.26	0.0023144	IHC/WB/IF
HOXB7	5.86	1.58	4.16	0.0088443	1.6	0.0886894	IHC
DLK1-DIO3 region							
SNORD113-7	0.19	ND	0.067	0.0019721	0.081	0.0258624	NA
SNORD113-5	0.17	ND	0.1	0.0021936	0.2868659	0.33	NA
SNORD112	0.11	ND	0.045	0.0148743	0.2573069	0.28	NA
SNORD114-31	0.2	ND	0.19	0.0904756	0.264131	0.27	NA
hsa-miR-134	ND	ND	0.38	0.0352731	ND	ND	NA
hsa-miR-382	ND	ND	0.14	0.0020194	ND	ND	NA
hsa-miR-544	ND	ND	0.37	0.0329153	ND	ND	NA
Cell proliferation and stemness related genes							
YY1	1.94	NA	21.26	0.0065292	7.24	0.0565113	IHC/WB/IP
MSX1	8.66	2.97	9.9	0.0344053	4.21	0.0149181	IHC
MKNK2	4.68	1.82	3.88	0.0055919	1.96	0.0075854	IHC/WB
TRIM71	4.4	NA	2.65	0.0044	1.85	0.0464	ND
C-MYC	1.1	1.34	0.744	0.75	0.52	0.109472	IHC/WB
HDAC2	1.35	0.9	1.53	0.84	0.92	0.32	IHC/WB/ Functional assay

Abbreviations: ND: not determined; NA: not applicable; IHC: immunohistochemistry; WB: western blotting; IF: immunofluorescence; IP: immunoprecipitation

# intragenic miRNA, in brackets the hosting gene

### Angiogenesis

#### Angiogenic factors up-regulated in ML: EGFL7 and EGFL6

EGFL7 is a protein that is almost exclusively expressed by endothelial cells and which, by means of NOTCH signalling, represses VEGFR pathway and stabilises developing vessels by maintaining the junctions between endothelial cells [11]. The vascular regulatory function of EGFL7 is reinforced by hsa-miR-126, which plays a role in correct vessel formation by modulating the response to growth factors (the VEGFA/VEGFR2 axis) and reducing the expression of endothelial cell adhesion molecules (V-CAM) [12-15]. In line with this involvement, hsa-miR-126 was down-modulated in RC as assessed by qRT-PCR (Table 1).

EGFL6 is a secreted protein whose receptor is not yet been defined. It is produced by mesenchymal cells [16], and promotes correct endothelial cell migration and tube formation by activating the ERK pathway [16]. It has been reported that its over-expression in tissue and serum correlates with benignity within the spectrum of meningiomas [17]. Like *EGFL7, EGFL6* harbours a miRNA (hsa-miR-6086) that was down-modulated in RC as assessed by qRT-PCR (Table 1), but whose role has not yet been fully clarified.

We were unable to obtain acceptable immunostaining for both of these factors, as often occurs when dealing with antibodies against ligands.

#### Angiogenic factors up-regulated in RC: Gremlin and *HOXB7*

Gremlin, which is known to act as an antagonist of BMP, may also function as a non-canonical VEGFR2 agonist [18] as it can induce the formation of the VEGFR2/αVß3 integrin complex that mediates paracrine/autocrine function in tumour vascularisation [19]. IHC showed that gremlin was expressed in the extra-cellular matrix, tumoral cells and vessels (Supplementary Figure S2 A,B) as previously described [20], and WB confirmed that it was more expressed in RC than in ML (Supplementary Figure S2 C). Confocal microscopy revealed the co-localisation of VEGFR2 and gremlin, a finding that suggests the gremlin-mediated enhancement of VEGFR2 activation (Supplementary Figure S2 panels D, E and F).

As regards *HOXB7*, we hypothesise that, in our context, this gene plays an oncogenic role by acting as a hub enhancing the activation of multiple factors (Supplementary Table S6). It has recently been demonstrated that the forced expression of HOXB7 in multiple myeloma induces the up-regulation of *VEGFA, FGF2, MMP2* and *PDGFA*, and the down-regulation of thrombospondin 2 [26], a profile largely shared by RC [38]. IHC analysis confirmed the molecular data by showing more tumoral cells with nuclear decoration in RC than in ML (Supplementary Figure S2 panels H and G), which suggests that HOXB7 plays a paracrine role although it may also be involved in regulating stemness.

Taken together, this evidence indicates that the progression from ML to RC is marked by changes that go from the “physiological-like” expression of proteins involved in regulating a proper vessel wall formation and function to the expression of proteins which, together with endothelial proteins and other factors, favour the development of an activated vascular phenotype [39].

### The *DLK1-DIO3* region

The DLK1-DIO3 genomic region located on human chromosome 14 (14q32) contains the paternally expressed imprinted genes *DLK1, DIO3* and *RTL1*, the maternally expressed imprinted long non-coding (lnc) RNA *MEG3* and *MEG8* genes and anti-sense *RTL1*, and one of the largest miRNA clusters in the genome: 54 miRNAs and 41 SNORDs.

This region is aberrantly expressed in most cancers and, although its function is not entirely understood, believed to possess tumour suppressor properties [40].

qRT-PCR confirmed microarray data showing that four SNORDs (SNORD 113-7, 113-5, 112 and 114-31) were more expressed in ML than in RC. Three miRNAs described to be located in this region (hsa-miR-134, hsa-miR-382 and hsa-miR-544) [40] were also assessed by qRT-PCR and found out again overexpressed in ML (Table 1).

However, when the region was analysed by means of FISH, no changes in copy number were found in either ML or RC (data not shown).

This evidence cumulatively supports the idea that the active state of the DLK1-DIO3 region in ML reduces cell proliferation and, as suggested by published data, that this epigenetic silencing is probably mediated by lnc RNA associated with chromatin-modifying complexes [41].

### Cell proliferation and stemness-related genes

Gene expression analysis and qRT-PCR indicated that genes involved in pluripotency and fast cell cycle (*YY1, MKNK2, MSX1* and *TRIM71*) were up-regulated in RC variant (Table 1). This segregation, coupled with the down-regulation of 14q32 region, strongly suggests that removing this restriction point is essential for resetting the epigenetic and transcriptional status of RC cells.

#### YY1 and YY1 interacting proteins

**Figure 3:**
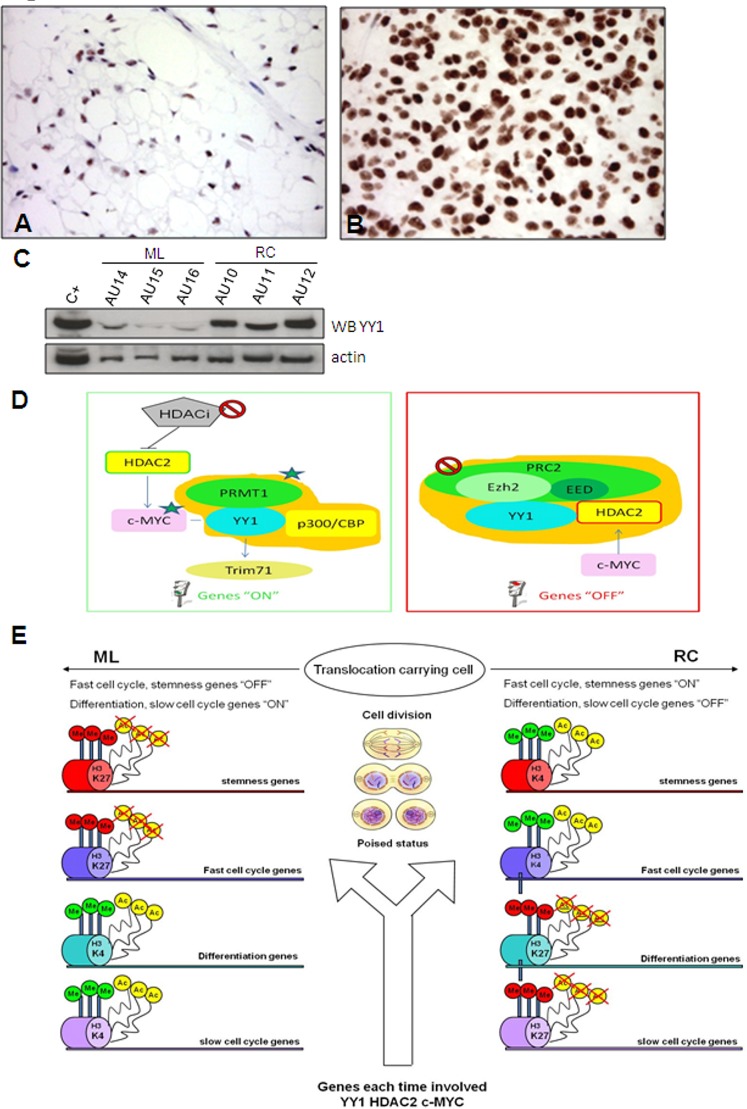
YY1. YY1 immunostaining shows faintly immunolabelled nuclei in the ML samples (A) and strong nuclear decoration in the RC samples involving the overwhelming majority of enriched tumoral cells (B). The results of the WB experiments were consistent with immunohistochemical results (C) Explanatory diagram of YY1 interactions. (D) The proposed model envisages mechanistic interactions between proteins and the promoters of the fast cell cycle/pluripotency/differentiation genes involved in MLS tumour progression. YY1 can activate (left) or repress (right) the transcription of its target genes. Left: Gene switch-on driven by YY1. YY1 triggers gene expression (“ON” genes) by recruiting histone acetyltransferases p300/CBP, which promote the histone acetylation and protein arginine methyltransferase PRMT 1 that lead to methylation at Arg3 of histone H4. The result is a high level of transcription activity supported by the hyper-acetylated and tri-methylated promoters shared by YY1 and c-MYC that leads to c-MYC network activation [32] involving fast cell cycle/pluripotency genes. Moreover, as c-MYC and HDAC2 play a mutually positive regulatory role, HDAC2 may activate c-MYC, thus making it a possible pharmacological target. TRIM71 is a downstream target that correlates significantly with YY1 (see Figure S3, described below). Right: Gene switch-off driven by YY1. PRC2 suppresses gene expression (“OFF” genes) by promoting histone H3 lysine 27 trimethylation (H3K27me3), and HDAC2 contributes to gene silencing by deacetylating histone. YY1 and HDAC2 physically interact and respectively bind PRC2 via Ezh2 and EED [28]. The result is silencing mediated by the deacetylation and tri-methylation of the promoters of the target genes involved in differentiation and fast cell cycle inhibition. Epigenetic modifications imposed by YY1, HDAC2 and c-MYC. (E) After each division, the fate of poised cells (translocation-carrying cells) is defined by genes targeted by the annotated proteins (YY1, HDAC2, c-MYC), which ensure progression to RC by means of protein-specific interplays that favour the repression or activation of the targeted genes in cooperation with chromatin-modifying complexes (thick arrow) of chromatin regulators (polycomb, trithorax, HDAC, HAC) and chromatin markers (H3K27, H3K4, histone deacetylation, histone acetylation). Polycomb and HDAC respectively induce gene silencing by means of H3K27 tri-methylation and histone deacetylation, whereas trithorax and HAC respectively trigger gene activation by means of H3K4 tri-methylation and histone acetylation. Cumulatively, the progression to RC is dictated by the enrichment of activated fast cell cycle/stemness genes and the silencing of differentiation/slow cell cycle genes.

YY1 over-expression in RC was confirmed by IHC (Figure 3A, B) and Western blotting (Figure 3C). YY1 is a transcription factor that modulates the transcription of other genes (Supplementary Figure S3, Supplementary Table S6) and acts by interacting with other proteins (Figure 3 D). In particular, it may interact with c-MYC (Figure 3D, left) and thus contribute to activating their shared target genes, and with HDAC2 (a PRC2 interacting protein) (Figure 3D, right) in order to suppress gene expression. In addition, c-MYC and HDAC2 have a mutual positive regulatory function.

#### c-MYC

**Figure 4:**
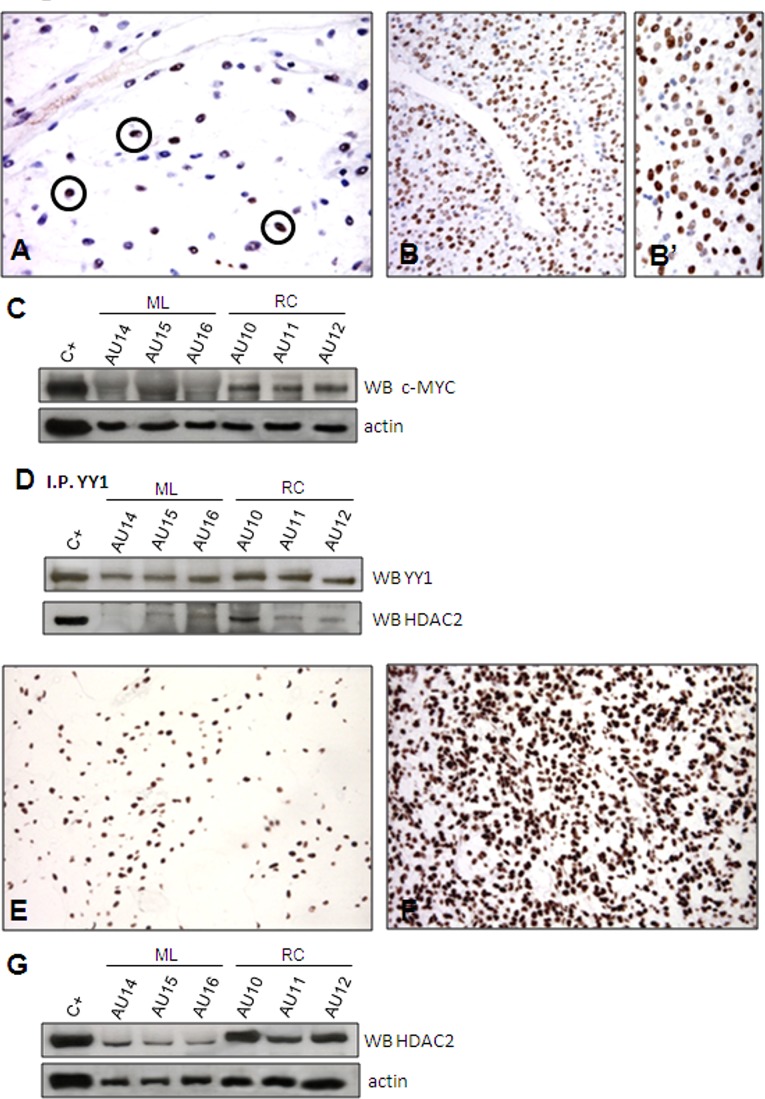
HDAC2. Only rare nuclei were immunoreactive for c-MYC in ML (A), whereas most of them were decorated in the RC samples (B, B'). The immunohistochemistry results were confirmed by WB (C). Co-IP experiment demonstrating a physical interaction between YYI and HDAC2 (D). Unlike the YYI- and c-MYC-immunolabelled samples, the HDAC2-immunolabelled samples showed nuclear decoration of the majority of tumoral cells in both ML (E) and RC (F). However, WB revealed HDAC2 over-expression in RC (G).

Despite the absence of *c-MYC* modulation at RNA level (Table 1), and given that YYI and c-MYC co-localise on the same promoters, that the over-expression of YY1 activates both endogenous and exogenous *c-MYC* promoters, and that the protein encoded by c-MYC can be post-translationally stabilised [42-44], we analysed c-MYC at protein level. Immunophenotyping revealed only rare, scattered c-MYC-immunolabelled nuclei in ML (Figure 4 A), whereas the overwhelming majority of nuclei were decorated in RC (Figure 4, B and B'), thus suggesting the post-translational stabilisation of C-MYC [44]. This result was confirmed by Western blotting (Figure 4C). IHC showed that c-MYC positivity correlated with an OCT4 and SOX2 null immunophenotype (data not shown), a signature that is consistent with that reported for c-MYC module [36] (Supplementary Table S6).

Taken together, these results suggest that cooperation between YY1 and c-MYC plays a pivotal role in triggering a specific RC network that favours a fast cell cycle (see Figure 3D left, and E).

### HDAC2

**Figure 5:**
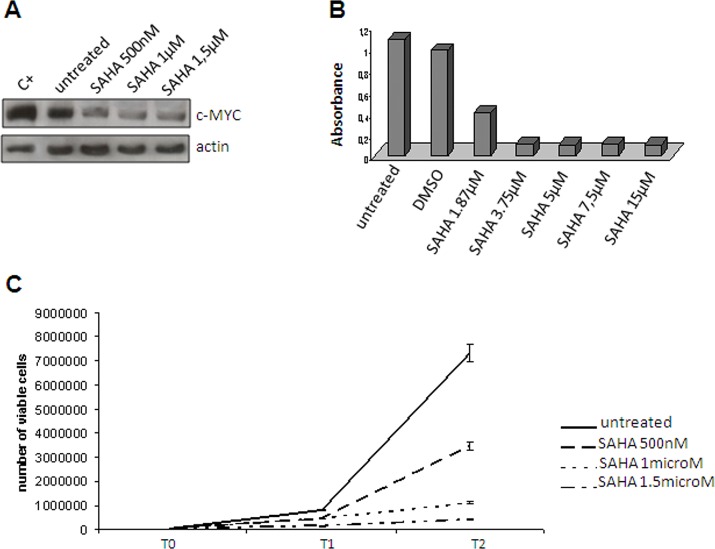
Vorinostat treatment Reduced c-MYC expression in the 402-91 cell line after vorinostat (SAHA) treatment. (A) MTT assay of the 402-91 cell line treated with vorinostat (SAHA). (B) Growth curve of the 402-91 cell line after three (T1) and six days of vorinostat (SAHA) treatment (T2) (C).

In order to substantiate our model further, we used HDAC2 as a surrogate of the PRC2 complex even though the gene expression analysis showed that it was not modulated (Table 1). HDAC2 is an additional partner of YY1 that has been reported to interact with YY1 physically [45]. YY1 and HDAC2 interact with PRC2 (respectively via Ezh2 and EED) to induce the silencing of differentiation-related genes (see Figure 3 D, right). After having confirmed the interaction between HDAC2 and YY1 by means of co-immunoprecipitation (Figure 4D), we used IHC (Figure 4A, B, B' and E, F) and Western blotting (Figure 4, C and G) of both c-MYC and HDAC2 (bearing in mind that c-MYC binds the promoter region of *HDAC2* and regulates the expression of PRC2 via HDAC2 regulation) [46, 47]. The readouts showed that the expression of c-MYC and HDAC2 was increased in RC, which is in line with a functional repressing role of PRC2. In order to confirm this result, we treated the 402-91 MLS cell line with vorinostat, an HDAC inhibitor [48, 49], and found a dose-dependent decrease in c-MYC protein (Figure 5A) and a reduction in cell growth (Figure 5, B and C).

Using HDAC2 as a surrogate to infer possible PRC2-induced silencing, we suggest that the YY1/c-MYC/HDAC2 axis plays a role in favouring stemness by modifying (via HDAC2) the chromatin-associated PRC2 that is known to silence differentiation-related genes, and provide evidence indicating a drug-induced slowing of proliferation.

#### MKNK2

The gene expression analysis showed that *MKNK2*, a kinase that specifically phosphorylates eukaryote translation initiation factor 4E (eIF4E) at Ser 209, was over-expressed in RC (Table 1), and this was confirmed by IHC and Western blotting (Supplementary Figure S4 A,A', B,B' and C). It has been demonstrated that eIF4E phosphorylation, which is dispensable for basal protein synthesis and cell viability, is necessary to increase the translation of the mRNAs involved in tumorigenesis, including *c-MYC* and *MCL1* [50, 51]. Consistently, and as previously shown in the case of c-MYC, MCL1 was over-expressed in RC as assessed by WB (Supplementary Figure S4D).

### MSX1

IHC showed that MSX1, over-expressed in RC at mRNA level (Table 1), is also increased at protein level (Supplementary Figure S5). MSX1 belongs to the HOX family, and its expression inhibits differentiation and thus leads to cells having properties similar to those of stem cells [52]. This inhibitory function is due to one of the most frequent mechanisms used by *HOX* genes to repress target genes: lnc RNA binding PRC2 (Supplementary Table S6 and Figure 3D). This is supported by the findings of recent studies of embryonic stem cells showing that MSX1 acts in competition with YYI and, by removing the PRC2/H3K27m3 repressor complex from YY1 and recruiting it to itself, represses MSX1 target genes [53, 54].

In our model, the co-overexpression of MSX1 and HDAC2 strongly suggests that they act together to force cells toward stemness.

#### TRIM71

Interrogation of the association between the expression profile of YYI and its downstream targets (Figure S4) showed that *TRIM71* is over-expressed in RC. TRIM71 is an RNA binding protein that is highly expressed in undifferentiated cells such as embryonic stem cells (ESCs) [55] and dynamically regulated during induced pluripotent stem cell (iPSC) reprogramming [55]), represses mRNAs regardless of the miRNA pathway [56] as well as modulates miRNA-mediated repression. It has been shown that TRIM71 functionally interacts with miRNA and the RISK complex which, as it contains Ago2 protein, can post-transcriptionally repress the expression of the cell cycle regulator Cdkn1a, and thus promote G1-S transition and the rapid proliferation of ESCs [57].

It has recently been suggested that TRIM71 belongs to the MYC module [55], thus supporting the idea that it may play a role in RC progression as a regulator of stemness and of the cell cycle.

Taken together, all of the above findings suggest that the make-up of the RC variant closely mirrors that of normal undifferentiated stem cells, and that YY1/c-MYC/HDAC2 axis, cell cycle-related MKNK2, stemness-related MSX1 and stemness/cell cycle-related TRIM71 are all involved in maintaining RC variant cells in a fast cycling and undifferentiated state.

### DISCUSSION

To the best of our knowledge, this is the first attempt to track a progression pathway from ML to RC liposarcomas by comparing the transcription profile of the two extremes of the tumour spectrum: the “pure forms” of the two variants. In this progression, the silencing of the DLK1-DIO3 region marks the transition to an epigenetic/transcriptional reprogramming, which is strongly aided by the involvement of genes promoting the fast cell cycle and pluripotency such as YY1 and YY1-associated genes, *MKNK2, MSX1* and *TRIM71*.

The shift from a “normal” phenotype to a pro-angiogenic signature probably represents the initial step in the progression from ML to RC, while epigenetic-based chromatin changes and the activation of transcriptional networks ultimately allow the cells to acquire (or re-enter) a stem-like state.

One novel finding of our study is the evidence indicating the significant up-regulation of the genes located on 14q32 region in ML that imposes the silencing of proliferation and pluripotency genes. The absence of any changes in copy numbers and the up-regulation of SNORDs and miRNAs revealed by qRT-PCR indicate the need to investigate this region further by focusing on epigenetic changes and miRNAs. It has recently emerged that the epigenetic regulation of tumour suppressor miRNAs is a critical signalling pathway involved in tumorigenesis [41]. It has been shown that miRNAs work closely with epigenetic modifiers, and that lnc RNAs may be physically associated with chromatin-modifying complexes for the purposes of activation (via histone tri-methylation or acetylation) or repression (via histone tri-methylation or deacetylation). Given the relatedness of the gene signature of embryonic stem and cancer cells [32, 36] and the fact that epigenetic modifications may change with each division of a cell carrying a tumoral translocation [58], we can imagine that, in our model, the chance of the daughter cell progressing to RC or remaining ML at each cycle greatly depends on the interplay of proteins (YY1, c-MYC and HDAC2) in concert with the enrichment of the activated fast cell cycle/stemness genes and the silenced differentiation/slow cell cycle genes in the case of RC, or *vice versa* in the case of ML (see Figure 3E). Although it needs to be re-examined by mean of focused high throughput technologies, this view also provides an explanation for the infrequent occurrence of pure MLS variants (about 0% or ≥80% of the round cell component).

After overcoming the barrier of the 14q32 region, it seems that changes in the expression of YY1 transcription factor and YY1-related genes are jointly responsible for driving cancer cells into their new cell state.

The apparently contradictory role played by YY1 (due to its ability to carry out different functions depending on the recruited co-factor) is also in line with the proposed model. Although the post-transcriptional interplay between YY1 and c-MYC and between YY1 and HDAC2 both favour tumour progression, the former activates the fast cell cycle/pluripotency genes, and the latter represses differentiation/fast cell cycle inhibitor genes. Consistently, the use of an HDAC inhibitor reduced cell growth.

In addition to c-MYC and MLC1, MKNK2 (a key gene in protein synthesis correlated with the fast cell cycle) was also differentially over-expressed in RC.

A stemness trait was given to the RC signature by the segregation of this variant with MSX1 over-expression, which was probably attributable to the competition between MSX1 and YY1 for PRC2.

Another marker of stemness that was significantly enriched and co-expressed with YY1 in RC was the *TRIM71* gene, which was detected by interrogating the association between YY1 expression and its downstream targets. In addition to stemness, it is thought that this gene also contributes to progression by promoting rapid proliferation via cdkn1 repression [57].

Taken together, the findings of this study strongly suggest that progression from ML to RC passes through a transition phase that in some way recapitulates the development/reprogramming of pluripotent stem cells in a human embryo [59] and that, in order to reset their new pathological stem-like differentiation signature, tumoral cells have to overcome the epigenetic silencing restriction site. This decommissioning seems to be a shared paradigm for tumours showing a spectrum of increasing malignancy [60-62] and epithelial-mesenchymal transition [63], and represents not only a prerequisite for progression towards higher grade/de-differentiated forms, but also a promising therapeutic target. The findings also suggest that the so-called “primitive” or “immature” translocation carrying cells of MLS should rather be regarded as “partially immature” as they acquire a robust stem-like signature when they progress from ML to RC.

Our gene discovery-based analysis only began to shed light on the multi-step process of ML-RC progression, which involves the complex interaction of genetic and epigenetic factors, but warrants further exploration.

## METHODS

### Ethics statement

Investigation has been conducted in accordance with the ethical standards and according to the Declaration of Helsinki and according to national and international guidelines and has been approved by the authors' institutional review board. In particular, the study was approved by the Independent Ethics Committee of the Fondazione IRCCS Istituto Nazionale dei Tumori di Milano (INT-MI). All of the patients whose biological samples were included in the study gave their signed consent to donate the tissues remaining after the diagnostic procedures had been completed at INT-MI.

### Patients and study design

A total of 24 patients undergoing the surgical resection of MLS were evaluated for this study. The sample areas chosen for the analysis contained more than 90% tumour cells, and no necrosis or normal tissue. The training series (INT-A) consisted of formalin-fixed paraffin-embedded (FFPE) surgical samples obtained from 12 patients treated at our institution between 1997 and 2009. The morphological characteristics of the pure myxoid and RC groups satisfied our selection criteria in all but one case (#AU19), which had a lower percentage of RCs. The validation series (INT-B) consisted of cryopreserved surgical samples obtained from 12 patients managed at our institution between 1978 and 2011; six cases were pure ML and six were pure RC.

Information about treatment and follow-up of the patients and molecular and molecular/cytogenetic characterisation of the samples are reported in Supplementary Methods.

Tables S1 and S2 summarise the clinical data, treatment, follow-up, and molecular cytogenetic characteristics of the two series for INT-A and INT-B respectively.

### Gene expression analysis

The microarrays were run as described in Supplementary Methods.

Total RNA from 10 μm sections of the FFPE INT-A specimens was isolated using the Qiagen RNeasy FFPE (Qiagen, Valencia, CA, USA) following the manufacturer's instructions, and was profiled on Illumina whole-genome DASL HumanHT-12 v4 BeadChips. Total RNA from the INT-B series was extracted from frozen samples using TriZol (Invitrogen), and was profiled on HumanHT-12_v4 BeadChips using the direct hybridisation assay.

All of the microarray data were MIAME compliant and the raw data were deposited into the NCBI's GEO database (http://www.ncbi.nmlm.nih.gov/projects/geo/) with accession number [GSE55466].

Quantitative reverse-transcription PCR (qRT-PCR) of gene and miRNA expression was performed as described in Supplementary Methods using the primers listed in Supplementary Table S3.

### Bioinformatic analysis

Class comparison, hierarchical clustering, global test of clustering, and heat map analyses were performed as described in Supplementary Methods. Sub-class mapping was carried out using the algorithm developed by Hoshida [9.].

### Immunohistochemistry (IHC)

Supplementary Table S4, part 1, shows the antibodies and experimental conditions used to detect gremlin, HOXB7, YY1, c-MYC, HDAC2, MKNK2 and MSX1.

### Immunofluorescence (IF) and confocal microscopy

The first part of the IF protocol was carried out as described for IHC in Supplementary Table S4. After being processed as previously described [3], the slides were incubated for 30 minutes at room temperature (RT) with rabbit anti-goat biotinylated antibody (E0466 DAKO, Carpinteria, CA) (dilution 1:100), followed by Alexa Fluor 546 streptavidin for one hour at RT (dilution 1:1000) (Invitrogen, Carlsbad, CA). Thereafter, the slides already incubated with anti-gremlin or VEGFR2 antibody (Supplementary Table S4, part 2), were incubated with Alexa Fluor 488 anti-rabbit antibody (Invitrogen) (dilution 1:1000) for one hour at RT. The rest of the protocol is described in Supplementary Methods.

### Western blotting (WB) and immunoprecipitation (IP)

The Western blotting analyses of gremlin, YY1, c-MYC, HDAC2 and MCL1 were made using 20 μg of protein extracts and the antibodies listed in Supplementary Table S4, part 3. Anti-actin antibody (1:2500; A2066; Sigma-Aldrich, St. Louis, MO) was used to ensure equal protein loading and to normalise the results.

The IP/WB analysis was made in order to evaluate the interaction between YY1 and HDAC2: equal amounts (300 μg) of protein lysates were precipitated by means of incubation with Protein A Sepharose (Amersham Biosciences, Piscataway, NJ) and 1 μl of anti-YY1 antibody. Anti-YY1 and HDAC2 antibodies (the same as those used for IHC) were used for the Western blotting.

### Vorinostat treatment of the myxoid liposarcoma cell line

Cell line 402-91, a recognised liposarcoma cell line [10], was treated with vorinostat (Cat. NoS1047-SAHA MK0683, Selleck Chemical, Houston, TX). The proliferation (MTT) and Trypan blue assays were carried out as described in Supplementary Methods. Western blotting of c-MYC was carried out using proteins extracted from untreated and treated cells under previously described conditions.

## SUPPORTING INFORMATIONS FIGURES AND TABLES














