# Supplementary material for: Identification of a gene expression driven progression pathway in myxoid liposarcoma
Source: Oncotarget. 2014 May 27;5(15):5965–77. doi: 10.18632/oncotarget.2023 (PMC4171605; doi:10.18632/oncotarget.2023)
Supplement: Supplementary file 1 [file oncotarget-05-5965-s001.pdf]

Identification of a gene expression driven progression pathway in myxoid liposarcoma

SUPPORTING INFORMATIONS

Treatment and follow-up

All the data are detailed in Tables S1 and S2.

Myxoid tumours. All but three of the patients with pure ML underwent surgery alone. RT was administered to three patients (INT-B) in whom the surgical margins were contaminated. At the time of the last contact, all of the patients were alive, and 8/12 were NED (no evidence of disease) (follow-up duration: 32-312 months).

Round cell tumours. All of the patients underwent surgery and RT and/or chemotherapy but two (one only surgery and one only RT). Three patients of the INT-A series and three of the INT-B developed metastases. The most frequent site of metastasis was lung, followed by bone and liver. All the patients of the INT-B series (but one lost) died within a range of 26 to 144 mos.

Molecular and molecular/cytogenetic characterisation

The diagnosis of MLS was confirmed by FISH and/or RT-PCR in all cases. Sanger sequencing showed 3/24 had *TP53* mutations (12.5%; two functional, and one non- functional), and two had *TP53* polymorphisms; 5/24 had *PIK3CA* mutations (20.8%); and 1/23 had a *PTEN* mutation (4.35%). Case AU28 (see Table S1) had both a non-functional *TP53* and a *PIK3CA* mutation. The percentages are in line with previously published data; there was no significant segregation of mutations with the ML or RC variants. For details, see Tables S1 and S2.

RNA extraction and gene expression microarray analysis

The microarrays were run at the Functional Genomics and Bioinformatics Core Facility of IRCCS Istituto Nazionale dei Tumori (Milan).

Total RNA was isolated using the Qiagen RNeasy FFPE kit (INT-A case material) or RNAeasy (INT-B case material) kit (Qiagen, Valencia, CA, USA). INT-A was profiled on Illumina whole-genome DASL HumanHT-12 v4 BeadChips containing probes for 29,285 transcripts (Illumina Inc., San Diego, CA). The DASL assay is a validated bead-based method that allows the expression profiling of RNA in FFPE samples. The experimental procedures, including labelling and hybridisation, were carried out following the manufacturer's protocol. Total RNA from the INT-B series was profiled on HumanHT-12_v4 BeadChips using the direct hybridisation assay, which allows the detection more than 47,000 transcripts. Briefly, 300 ng of total RNA was reverse transcribed, labelled with biotin, and amplified overnight using the Illumina RNA TotalPrep Amplification kit (Ambion); 1 ug of the biotinylated cRNA sample was hybridised to the BeadChips at 58°C overnight. After washing, the array were stained with 1 ug/mL Cy3-streptavidine and scanned with Illumina BeadArray Reader.

In both cases, the images were analysed and the primary data were collected using the supplied scanner software. The average intensity of all of the beads belonging to a single probe was calculated with its P value using BeadStudio software v3 and BeadStudio expression analysis module v3.3.8; the data were quantile normalised and exported without correcting for background or scale. The data matrices were filtered in order to exclude signals with detection P values of >0.05 and allow 50% of missing values.

Bioinformatics analysis

The differentially expressed genes were defined by imposing an FDR of <10%. The significance of the individual genes was tested by means of the univariate permutation test using BrB ArrayTools_v4.1.0-stable release (Simon, R. and Lam, AP; http://linus.nci.nih.gov/BRB-ArrayTools.html).

The sub-class mapping algorithm (Gene Pattern Software, Version 3.0; Broad Institute) [9] was used to ascertain whether the pattern of the modulated genes in the training set molecularly corresponded to that observed in the validation set. This algorithm computes the enrichment of each predefined phenotype between the first and second dataset, and also provides a p-value. Sub-class mapping has the advantage of being an unsupervised method for identifying molecular similarity in groups of samples from independent datasets beyond technical differences.

Quantitative reverse-transcription PCR (qRT-PCR) of gene and miRNA expression

The gene and miRNA primers were respectively purchased from Applied Biosystems (Foster City, CA, USA), and Exiqon (Vedbæk, Denmark), except for the primers for *MSX1*, which were custom designed on the basis of the *MSX1* refseq (NM_002448) and purchased from IDT (Integrated DNA Technologies, Inc, CA, USA). The assay IDs are listed in Table S2.

Gene expression qRT-PCRs were performed using a TaqMan transcription kit (Applied Biosystem, Foster City, CA), and real-time PCRs using the TaqMan Gene Expression Master Mix, TaqMan MicroRNA assays (Applied Biosystems) or TaqMan gene expression assays (Applied Biosystems). The reactions were incubated for 10 minutes at 95°C, followed by 40 cycles of 15 seconds at 95°C and one minute at 60°C. The qRT-PCR data shown in Table1 were normalised using the RPL13a housekeeping gene; similar results were obtained testing a second housekeeping gene (β-actin).

The miRNA qRT-PCRs were performed using the miRCURY LNA Universal RT microRNA PCR system (Exiqon) in accordance with the manufacturer's instructions. Total RNA (20 ng) was polyadenylated and reverse-transcribed at 42°C (60 min), and then heat-inactivated at 85°C (5 min) using a poly-T primer containing a 5′ universal tag. The resulting cDNA was diluted 80-fold, and 8 µL was used in 20 µL PCR amplification reactions at 95°C for 10 min, 40 cycles of 95°C for 10 sec, and 60°C for 60 sec. The results were normalised using snord48 (Assay ID:203903).

The expression levels were quantified using a sequence detection system (QuantStudio; LifeTechnologies) in triplicate, and the threshold cycle (Ct) was determined for each sample. Relative miRNA and mRNA expression was analysed using the ΔΔCT method.

Immunofluorescence (IF) and confocal microscopy

After washing three times with 0.01% (v/v) Triton X-100 in PBS buffer, the slides were mounted using Vectashield mounting medium with DAPI (H1200, Vector Labs, Burlingame, CA), and analysed by means of a confocal microscope (Microradiance 2000, Bio-Rad Laboratories, Inc., Hercules, CA) equipped with Ar (488 nm), HeNe (543 nm) and red diode lasers (637 nm). The images (512x512 pixels) were acquired as previously described [3].

Vorinostat treatment of the myxoid liposarcoma cell line

Vorinostat (Cat NoS1047-SAHA MK0683 Selleck Chemical) was resuspended at 150 mM in DMSO, and diluted to final concentrations ranging from 500 nM to 15 μM) in RPMI cell culture medium (maximum DMSO concentration 0.0005%).

Proliferation assay (MTT)

$1.5x10^3$ cells were seeded in a flat-bottomed 96-well plates in a volume of 200 μl of RPMI cell medium. After 24 hours, cells were treated with vorinostat at final concentration ranging from 1.87 μM to 15 μM. After 72 hours of culture, 20 μl of 5mg/L MTT (Cat M5655, Sigma Aldrich, St. Luis, MO) were added directly to the cells followed by an additional 4 hours incubation, and then 100 μl DMSO were added. The adsorbance of individual wells was read at a wavelength of 550nm.

Trypan blue assay (growth curve)

$3x10^5$ cells were seeded in 10x20 mm tissue culture dish (cat. 35503, Becton Dikinson). Vorinostat was added at final concentration ranging from 500 nM to 1.5 μM in RPMI cell culture medium. After 3 and 6 days, the cells were detached using a 1% trypsin-EDTA solution (Cat. No. 15400, Invitrogen) and counted using a Burker chamber.

c-MYC WB

Proteins were extracted from vorinostat-treated cells and immunoblotted with c-MYC antibody as described above.

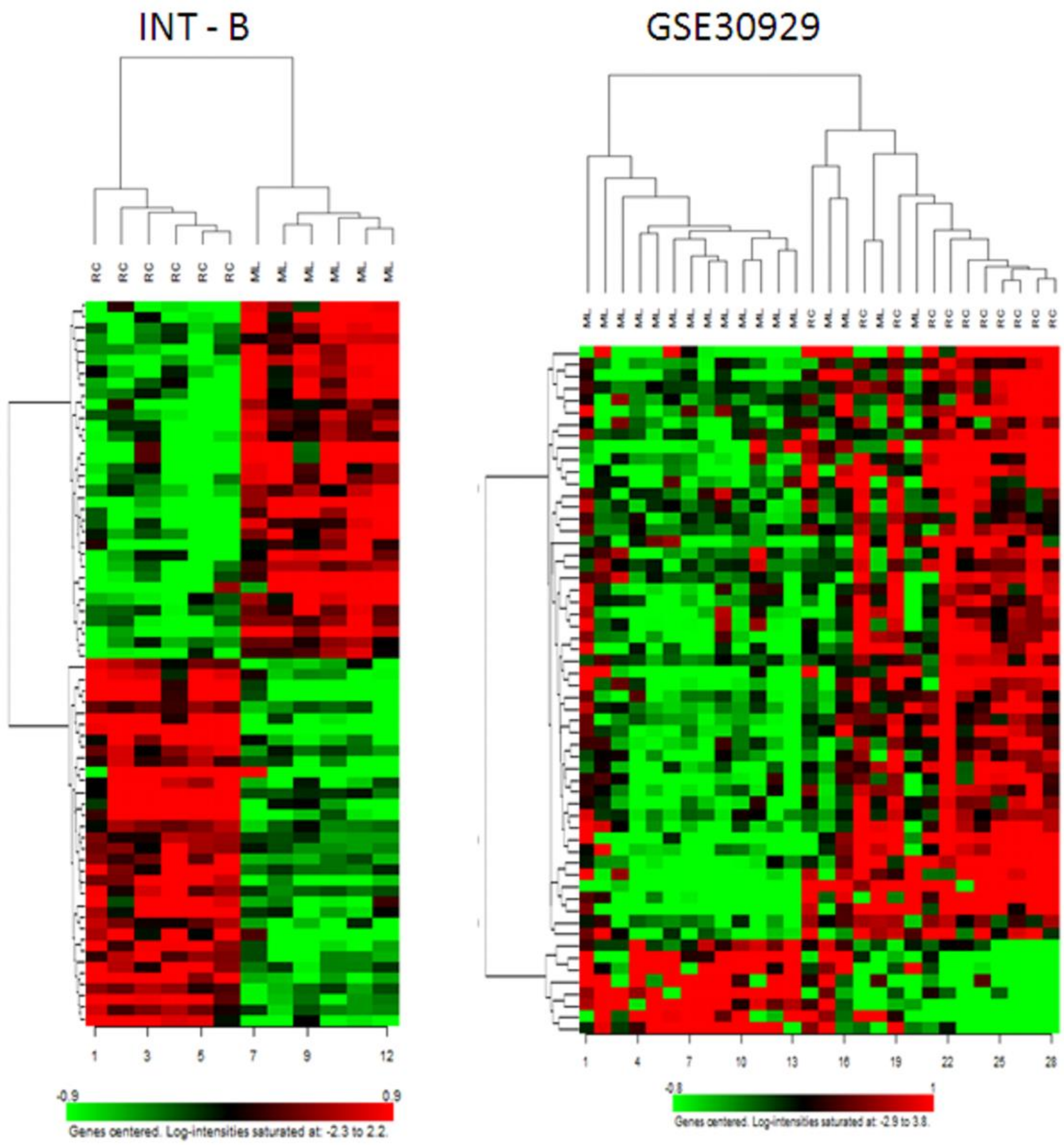

Figure S1: Heatmap of the genes differentially expressed in INT-B and GSE30929 after imposing an FDR of <0.1.

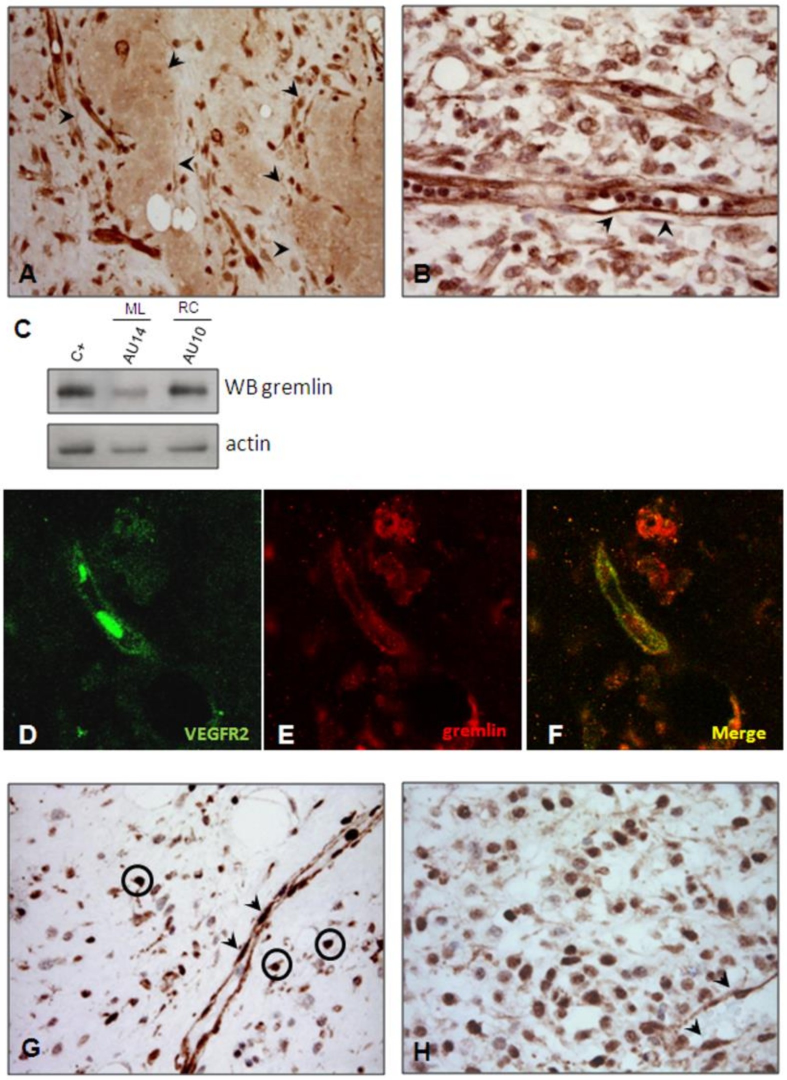

Figure S2: Gremlin and HOXB7. In line with the fact that gremlin exists in secreted and cell-associated forms, it decorated the extracellular matrix in ML (A, arrowheads), and the parenchymal cell and the heparin sulfate proteoglycans of the endothelial surface in both ML and RC (B, arrowheads) [21]. WB showing gremlin over-expression in RC. C) Co-localisation of VEGFR2 (D, green signal) and gremlin (E, red signal) was demonstrated by immunofluorescence (F, yellow signal). Immunodecoration of HOXB7 (G, H). The nuclei of tumoral and endothelial cells (arrowheads) were decorated. Note the difference in the proportion of immunolabelled nuclei of tumoral cells between ML (circled)(G) and RC (H).

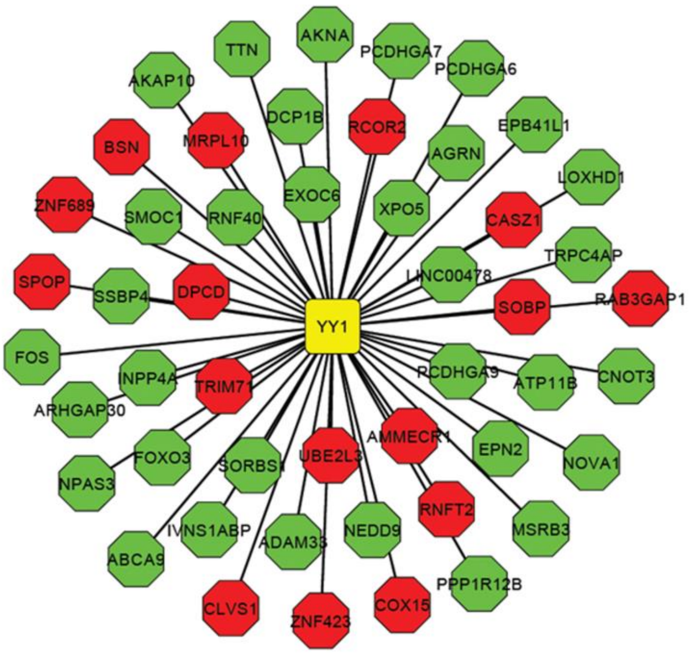

Figure S3: Computational integration of YY1 and target genes. MAGIA was used to correlate with the expression of YY1 with that of its transcriptional targets, and the 49 detected interactions were visualised in the network generated by Cytoscape. Red = positive correlations, and green = negative correlations.

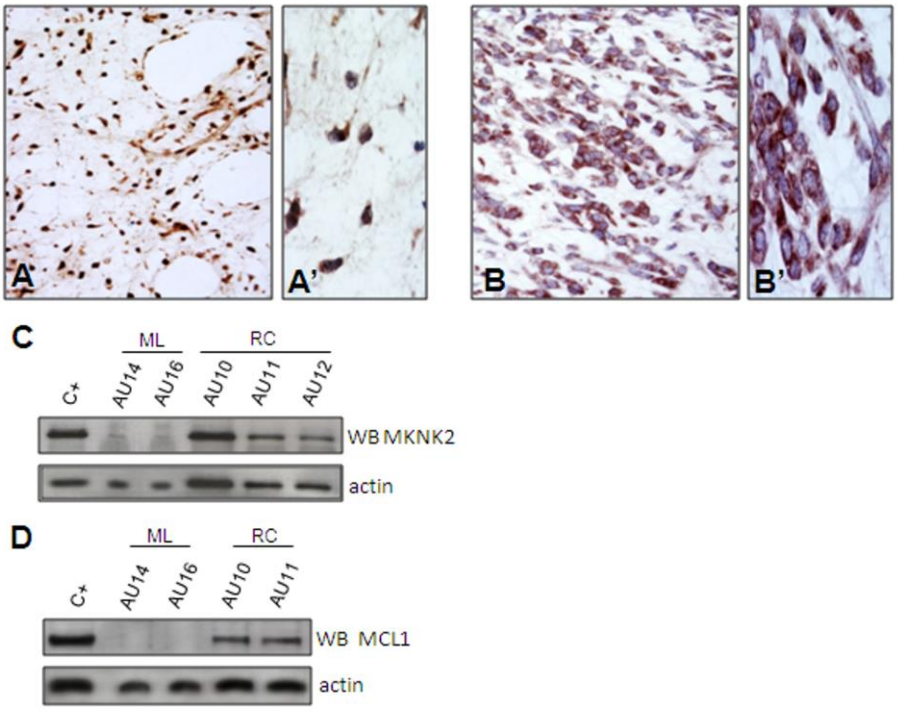

Figure S4: MKNK2. Increasing of immunolabeled cytoplasms of tumoral cells running from ML (A; A' higher magnification) to RC (B; B' higher magnification). C) WB confirmed the immunophenotyping results. Note that MCL1 had a similar WB read out (D): this is in line with the specific role of MKNK2 in phosphorylating 4E, which increases the translation of the mRNAs involved in tumorigenesis to which *MLC1* belongs.

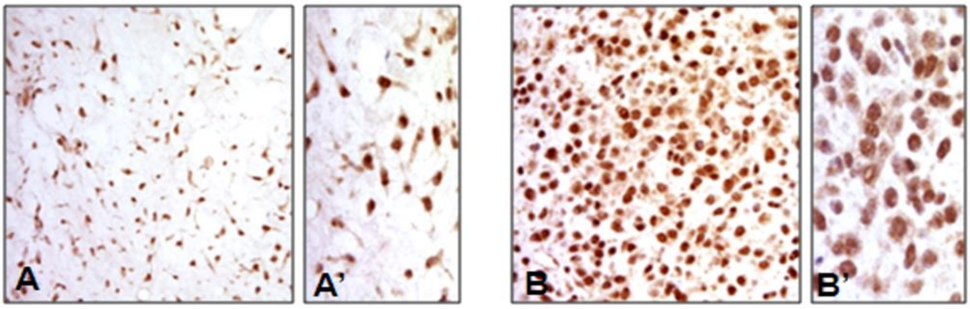

Figure S5: MSX1. The MSX1-immunolabelled samples showed a marked increase in decorated nuclei moving from ML (A; A’ higher magnification) to the RC variant (B; B’ higher magnification).
