# Supplementary material for: Identification of a gene expression driven progression pathway in myxoid liposarcoma
Source: Oncotarget. 2014 May 27;5(15):5965–77. doi: 10.18632/oncotarget.2023 (PMC4171605; doi:10.18632/oncotarget.2023)
Supplement: Supplementary file 2 [file oncotarget-05-5965-s002.doc]

| **Supplementary Table S1** | | | |  |  |  |  |  |  |  |  |  |  |
| --- | --- | --- | --- | --- | --- | --- | --- | --- | --- | --- | --- | --- | --- |
| **INT-A** | |  | | | | | | | | | | | |
|  | | | | | | | | | | | **Sequencing status** | | |
| **ID GF** | **Gender / Age** | **Primary Site** | **Tumor subtype** | **reccurrence number** | **time of 1^ reccurrence (mos)** | **time of 1^metastasis**  **(mos)** | **metastasis site** | **treatment** | **Disease state F.up duration ( mos)** | **DDIT3 rearrangement by FISH / fusion type by RT-PCR** | **TP53** | **PIK3CA** | **PTEN** |
| AU17 | ♀ / 54 | thigh | ML | 3 | 170 |  |  | surgery | A, NED, 312 | DDIT3 rearrangement | wt | wt | ND |
| AU21 | ♂ / 51 | thigh | ML | 3 | 36 |  |  | surgery | A, NED, 200 | FUS-DDIT3 type II | wt | wt | wt |
| AU23 | ♀ / 34 | thigh | ML |  |  |  |  | surgery | A, NED, 124 | FUS-DDIT3 type I | wt | H1047R | wt |
| AU29 | ♀ / 33 | thigh | ML |  |  |  |  | surgery | A, NED, 106 | DDIT3 rearrangement | wt | wt | wt |
| AU24 | ♀ / 56 | thigh | ML |  |  |  |  | surgery | A, NED, 75 | DDIT3 rearrangement | wt | wt | wt |
| AU25 | ♀ / 82 | thigh | ML | 4 | 24 |  |  | surgery | A, NED, 94 | DDIT3 rearrangement | wt | wt | wt |
| AU30 | ♂ / 52 | leg | RC 100% | 1 | 21 | 72 | lung bone | surgery/ CT/ trabectedin | D, WD, 97 | DDIT3 rearrangement | wt | wt | wt |
| AU27 | ♀ / 47 | bottock | RC>80% | 1 | 4 |  |  | surgery/ RT/CT | A, NED, 61 | DDIT3 rearrangement | functional A129T | H1047R | wt |
| AU28 | ♂ / 42 | shoulders | RC 80% |  |  | 46 | lung | surgery/RT | A, NED, 61 | DDIT3 rearrangement | no functional Y163C | H1047R | wt |
| AU22 | ♂ / 39 | thigh | RC 90% |  |  |  |  | surgery/ RT/CT | A, NED, 63 | DDIT3 rearrangement | wt | wt | wt |
| AU26 | ♀ / 58 | thigh | RC 80% |  |  | 16 | lung | surgery/RT | A, NED, 68 | DDIT3 rearrangement | wt | wt | wt |
| AU19 | ♂ / 61 | thigh | RC 15% |  |  |  |  | surgery | A, NED, 66 | DDIT3 rearrangement | wt | wt | C136Y |
