# Supplementary material for: Identification of a gene expression driven progression pathway in myxoid liposarcoma
Source: Oncotarget. 2014 May 27;5(15):5965–77. doi: 10.18632/oncotarget.2023 (PMC4171605; doi:10.18632/oncotarget.2023)
Supplement: Supplementary file 3 [file oncotarget-05-5965-s003.doc]

| **Supplementary Table S2** | | | |  |  |  |  |  |  |  |  |  |  |
| --- | --- | --- | --- | --- | --- | --- | --- | --- | --- | --- | --- | --- | --- |
| **INT-B** | |  | | | | | | | | | | | |
|  | | | | | | | | | | | **Sequencing status** | | |
| **ID GF** | **Gender / Age** | **Primary Site** | **Tumor subtype** | **reccurrence number** | **time of 1^ reccurrence (mos)** | **time of 1^metastasis**  **(mos)** | **metastasis site** | **treatment** | **Disease state F.up duration ( mos)** | **DDIT3 rearrangement by FISH / fusion type by RT-PCR** | **TP53** | **PIK3CA** | **PTEN** |
| AU06 | ♀ /33 | pelvis | ML | 12 | 36 |  |  | surgery RT/CT | A, WD, 168 | FUS-DDIT3 type II | wt | wt | wt |
| AU05 | ♂ / 42 | groin | ML | 8 | 36 |  |  | surgery RT/CT | A, WD, 272 | FUS-DDIT3 type II | wt | wt | wt |
| AU07 | ♀ / 34 | thigh | ML | 2 | 170 |  |  | surgery | A, NED, 300 | FUS-DDIT3 type II | wt | wt | wt |
| AU16 | ♂ / 60 | arm | ML |  |  |  |  | surgery | A, NED, 51 | DDIT3 rearrangement | no functional R280K | wt | wt |
| AU14 | ♀ / 42 | thigh | ML |  |  |  |  | surgery | A, NED, 41 | DDIT3 rearrangement | wt | wt | wt |
| AU15 | ♂ / 81 | leg | ML |  | 12 |  |  | surgery/RT | A, NED, 32 | DDIT3 rearrangement | wt | H1047R | wt |
| AU08 | ♂ / 62 | thigh | RC 80% | 1 | 24 | 24 | lung | surgery RT/CT | DOD, 26 | FUS-DDIT3 type II | polymorph264 | H1047R | wt |
| AU09 | ♂ / 47 | thigh | RC 80% | 2 | 12 | 24 | lung | surgery/CT | DOD, 46 | FUS-DDIT3 type II | wt | wt | wt |
| AU10 | ♀ / 51 | thigh | RC 80% | 2 | 60 |  |  | surgery RT/CT trabectedin | DOD, 144 | DDIT3 rearrangement | wt | wt | wt |
| AU11 | ♂ / 31 | leg | RC 80% |  |  |  |  | RT | LOST | FUS-DDIT3 type I | wt | wt | wt |
| AU12 | ♂ / 59 | bottock | RC 80% | 2 | 12 | 36 | liver bone (spine) | surgery/CT | DOD, 41 | DDIT3 rearrangement | polymorph213 | wt | wt |
| AU13 | ♀ / 43 | thigh | RC 80% | 3 | 12 |  |  | surgery/RT | DOD, 52 | FUS-DDIT3 type II | wt | wt | wt |
