# Supplementary material for: Identification of a gene expression driven progression pathway in myxoid liposarcoma
Source: Oncotarget. 2014 May 27;5(15):5965–77. doi: 10.18632/oncotarget.2023 (PMC4171605; doi:10.18632/oncotarget.2023)
Supplement: Supplementary file 4 [file oncotarget-05-5965-s004.doc]

| Supplementary Table S3 | |
| --- | --- |
| **gene_symbol** | **Taqman** |
| EGFL6 | **Hs01556006_m1** |
| EGFL7 | **Hs00211952_m1** |
| GREM2 | **Hs03986140_s1** |
| MKNK2 | **Hs00179671_m1** |
| HOXB7 | **Hs04187556_m1** |
| SNORD112 | **Hs03298810_s1** |
| SNORD113-5 | **Hs03299144_s1** |
| SNORD113-7 | **Hs03299146_s1** |
| SNORD114-31 | **Hs03299132_s1** |
| c-MYC | **Hs99999003_m1** |
| YY1 | **Hs00231533_m1** |
| HDAC2 | **Hs00231032_m1** |
| TRIM71 | **Hs01394933_m1** |
|  |  |
| ACTB | **Hs01060665_g1** |
| RPL13A | **Hs01926559_g1** |
|  |  |
| **gene_symbol** | **Assay from IDT** |
| MSX1 | **NM_002448** |
|  |  |
| **miRNA** | **Exiqon** |
| hsa-miR-126 | **204227** |
| hsa-miR-134 | **205896** |
| hsa-miR-382 | **204169** |
| hsa-miR-544 | **204646** |
| hsa-miR-6086 | **custom design** |
|  |  |
| snord48 | **203903** |
