# Supplementary material for: Identification of a gene expression driven progression pathway in myxoid liposarcoma
Source: Oncotarget. 2014 May 27;5(15):5965–77. doi: 10.18632/oncotarget.2023 (PMC4171605; doi:10.18632/oncotarget.2023)
Supplement: Supplementary file 5 [file oncotarget-05-5965-s005.doc]

| **TABLE S4: Methodological conditions.** | | | | |
| --- | --- | --- | --- | --- |
| **Part 1** |  | | | |
|  | | | **IHC condition** | |
| **Antibody** | **Clone** | **Company** | **Dilution** | **Antigen retrieval** |
| Gremlin | MAB3047 | R&D | 1:50 | citrate buffer pH 6, 40' at 95°C, overnight at 4°C |
| HOXB7 | SAB1412036 | Sigma Aldrich | 1:100 | benchmarkULTRA ventana,standard,CC1 buffer, optiviewDAB |
| YY1 | ab109237 | abcam | 1:250 | benchmarkULTRA ventana,standard,CC1 buffer, optiviewDAB |
| c-MYC | ab32072 | abcam | 1:50 | autostainerlink48 DAKO, high 30'+ LR |
| HDAC2 | ab32117 | abcam | 1:200 | benchmarkULTRA ventana,standard,CC1 buffer, optiviewDAB |
| MKNK2 | ab84345 | abcam | 1:100 | benchmarkULTRA ventana,standard,CC1 buffer, ultraviewDAB |
| MSX1 | MAB5045 | R&D | 1:10 | citrate buffer pH 6, 7' at 95°C |
| **Part 2** |  | | | |
|  | | | **IF condition** | |
| **Antibody** | **Clone** | **Company** | **Dilution** | **Antigen retrieval** |
| Gremlin | MAB3047 | R&D | 1:50 | EDTA buffer pH 8, 15' at 95°C, overnight at 4°C |
| VEGFR2 | #2479 | CellSignalling | 1:300 | EDTA buffer pH 8, 15' at 95°C, overnight at 4°C |
| **Part 3** | | |  |  |
|  | | | **WB condition** | |
| **Antibody** | **Clone** | **Company** | **Dilution** | |
| Gremlin | MAB3047 | R&D | 1:2000 BSA 5% | |
| YY1 | ab109237 | abcam | 1:1000 BSA 5% | |
| c-MYC | ab32072 | abcam | 1:10000 BSA 5% | |
| HDAC2 | ab32117 | abcam | 1:1000 BSA 5% | |
| MKNK2 | ab84345 | abcam | 1:1000 BSA 5% | |
| MCL1 | #4572 | CellSignalling | 1:1000 BSA 5% | |
