# Supplementary material for: Identification of a gene expression driven progression pathway in myxoid liposarcoma
Source: Oncotarget. 2014 May 27;5(15):5965–77. doi: 10.18632/oncotarget.2023 (PMC4171605; doi:10.18632/oncotarget.2023)
Supplement: Supplementary file 6 [file oncotarget-05-5965-s006.rtf]

Supplementary Table S5
Unique id
GB acc
Gene symbol
EntrezID
p-value
FDR
Permutation
p-value
log2(fold-
change)
Cytoband
GO
ILMN_2347068
NM_017572
MKNK2
2872
0
.0
0
0
0
1
5
9
0
.0
2
8
6
0
.0
0
0
2
1
6
4
5
2
.2
2
6
5
0
8
5
3
1
9
p13.3
ATP binding|metal ion binding|nucleotide binding|protein serine/threonine kinase
activity|apoptotic process|cell surface receptor signaling pathway|cellular response to
arsenic-containing substance|hemopoiesis|intracellular protein kinase cascade|protein
phosphorylation|regulation of translation|PML body|cytoplasm|nucleus
ILMN_2309156
NM_199169
PMEPA1
56937
0
.0
0
0
0
1
8
1
0
.0
2
8
6
0
.0
0
0
2
1
6
4
5
-
0
.9
4
3
4
1
6
4
7
2
2
0
q13.31-q13.33
WW domain binding|molecular_function|androgen receptor signaling pathway|integral to
membrane|plasma membrane
ILMN_1794803
NM_000266
NDP
4693
0
.0
0
0
0
3
4
9
0
.0
2
8
6
0
.0
0
0
2
1
6
4
5
3
.1
9
8
4
9
4
1
5
4
Xp11.4
cell surface binding|cytokine activity|frizzled binding|growth factor activity|protein
homodimerization activity|Wnt receptor signaling pathway|canonical Wnt receptor signaling
pathway|cell proliferation|cell-cell signaling|extracellular matrix-cell signaling|nervous
system development|placenta development|positive regulation of sequence-specific DNA
binding transcription factor activity|positive regulation of transcription, DNA-
dependent|response to stimulus|retina vasculature morphogenesis in camera-type
eye|sensory perception of sound|signal transduction|vacuole organization|visual
perception|extracellular matrix|extracellular region|extracellular space
ILMN_2347541
NM_182945
NIN
51199
0
.0
0
0
0
5
4
5
0
.0
3
1
7
0
.0
0
0
2
1
6
4
5
-
0
.8
6
2
4
9
6
4
7
6
ILMN_1735959
NM_013316
CNOT4
4850
0
.0
0
0
0
6
2
7
0
.0
3
2
1
0
.0
0
0
2
1
6
4
5
-
0
.9
1
5
9
3
5
7
3
5
7
q22-qter
RNA binding|ligase activity|metal ion binding|nucleotide binding|protein binding|ubiquitin-
protein ligase activity|zinc ion binding|RNA metabolic process|gene expression|mRNA
metabolic process|nuclear-transcribed mRNA catabolic process, deadenylation-dependent
decay|nuclear-transcribed mRNA poly(A) tail shortening|protein
autoubiquitination|regulation of transcription, DNA-dependent|cytoplasm|cytosol|nucleus
ILMN_2340565
NM_001001486
ATP2C1
27032
0
.0
0
0
0
7
1
5
0
.0
3
2
9
0
.0
0
0
2
1
6
4
5
-
0
.8
6
2
4
9
6
4
7
6
3
q22.1
ATP binding|ATP binding|calcium ion binding|calcium-transporting ATPase activity|calcium-
transporting ATPase activity|calcium-transporting ATPase activity|calcium-transporting
ATPase activity|hydrolase activity|hydrolase activity, acting on acid anhydrides, catalyzing
transmembrane movement of substances|manganese ion binding|manganese-transporting
ATPase activity|metal ion binding|nucleotide binding|signal transducer activity|ATP
biosynthetic process|Golgi calcium ion homeostasis|Golgi calcium ion transport|actin
cytoskeleton reorganization|calcium ion transport|calcium ion transport|calcium-dependent
cell-cell adhesion|cation transport|cellular calcium ion homeostasis|cellular calcium ion
homeostasis|cellular manganese ion homeostasis|epidermis development|ion
transmembrane transport|manganese ion transport|positive regulation of I-kappaB
kinase/NF-kappaB cascade|signal transduction|transmembrane transport|Golgi
apparatus|Golgi membrane|Golgi membrane|integral to membrane|membrane|trans-Golgi
network
ILMN_2173500
NM_001044369
C18orf51
125704
0
.0
0
0
0
7
2
1
0
.0
3
2
9
0
.0
0
0
2
1
6
4
5
2
.1
3
0
9
3
0
8
7
1
8
q22.3
endoplasmic reticulum|endoplasmic reticulum membrane|integral to membrane|membrane
ILMN_2119297
NM_015589
SAMD4A
23034
0
.0
0
0
0
8
0
1
0
.0
3
5
5
0
.0
0
0
2
1
6
4
5
-
2
.1
8
4
4
2
4
5
7
1
1
4
q22.2
translation repressor activity|negative regulation of translation|positive regulation of
translation|cell junction|cell projection|cytoplasm|dendrite|synapse|synaptosome
ILMN_1678816
NM_022469
GREM2
64388
0
.0
0
0
0
8
2
3
0
.0
3
5
5
0
.0
0
0
2
1
6
4
5
2
.4
2
4
9
2
2
0
8
8
1
q43
cytokine activity|BMP signaling pathway|extracellular region|extracellular space
ILMN_1716309
NM_015507
EGFL6
25975
0
.0
0
0
1
1
5
5
0
.0
4
4
0
.0
0
0
2
1
6
4
5
-
3
.8
7
8
3
2
1
4
4
3
Xp22
calcium ion binding|integrin binding|cell adhesion|cell cycle|cell differentiation|multicellular
organismal development|basement membrane|extracellular region|extracellular
space|membrane                                              


ILMN_1803956
NM_033254
BOC
91653
0
.0
0
0
1
1
7
5
0
.0
4
4
0
.0
0
0
2
1
6
4
5
-2
3
q13.2
protein binding|cell adhesion|muscle cell differentiation|positive regulation of muscle cell
differentiation|positive regulation of myoblast differentiation|integral to membrane|plasma
membrane
ILMN_1689177
NM_001030059
PPAPDC1A
196051
0
.0
0
0
1
2
6
9
0
.0
4
6
2
0
.0
0
0
2
1
6
4
5
-
3
.6
0
8
2
3
2
2
8
1
0
q26.12
hydrolase activity|phosphatidate phosphatase activity|phospholipid
dephosphorylation|integral to membrane|membrane
ILMN_1710523
NM_005603
ATP8B1
5205
0
.0
0
0
1
5
8
2
0
.0
4
8
5
0
.0
0
0
2
1
6
4
5
-
2
.6
4
3
8
5
6
1
9
1
8
q21-q22|18q21.31
ATP binding|ATPase activity|ATPase activity, coupled to transmembrane movement of ions,
phosphorylative mechanism|hydrolase activity|hydrolase activity, acting on acid anhydrides,
catalyzing transmembrane movement of substances|magnesium ion binding|nucleotide
binding|phospholipid-translocating ATPase activity|protein binding|ATP catabolic
process|bile acid and bile salt transport|bile acid metabolic process|cation transport|ion
transmembrane transport|negative regulation of transcription, DNA-
dependent|phospholipid translocation|transmembrane transport|apical plasma
membrane|brush border membrane|endoplasmic reticulum|integral to plasma
membrane|membrane fraction|plasma membrane
ILMN_3238950
NR_003080
SNORD112
692215
0
.0
0
0
2
0
2
0
.0
4
8
5
0
.0
0
0
5
4
1
1
2
5
-
3
.1
8
4
4
2
4
5
7
1
ILMN_2315979
NM_030915
LBH
81606
0
.0
0
0
2
4
0
6
0
.0
5
8
8
0
.0
0
0
2
1
6
4
5
-
2
.3
9
5
9
2
8
6
7
6
2
p23.1
multicellular organismal development|positive regulation of transcription, DNA-
dependent|cytoplasm|intracellular membrane-bounded organelle|nucleolus|nucleus
ILMN_1671843
NM_001032290
PSRC1
84722
0
.0
0
0
2
5
2
7
0
.0
6
0
5
0
.0
0
0
2
1
6
4
5
2
.0
0
7
1
9
5
5
0
1
ILMN_2256295
NM_012483
GNLY
10578
0
.0
0
0
2
5
6
4
0
.0
6
0
5
0
.0
0
0
8
6
5
8
-
3
.0
5
8
8
9
3
6
8
9
2
p11.2
ILMN_1684401
NM_002021
FMO1
2326
0
.0
0
0
2
5
8
5
0
.0
6
0
5
0
.0
0
0
2
1
6
4
5
-2
1
q24.3
N,N-dimethylaniline monooxygenase activity|N,N-dimethylaniline monooxygenase
activity|NADP binding|flavin adenine dinucleotide binding|monooxygenase activity|NADPH
oxidation|organic acid metabolic process|response to lipopolysaccharide|response to
osmotic stress|small molecule metabolic process|toxin metabolic process|xenobiotic
metabolic process|endoplasmic reticulum|endoplasmic reticulum lumen|integral to
membrane|intrinsic to endoplasmic reticulum membrane|membrane|microsome
ILMN_1791222
NM_145262
GLYCTK
132158
0
.0
0
0
2
9
5
7
0
.0
6
3
2
0
.0
0
0
4
3
2
9
-
1
.8
3
6
5
0
1
2
6
8
3
p21.1
ATP binding|glycerate kinase activity|nucleotide binding|protein binding|transferase
activity|protein phosphorylation|Golgi apparatus|cytoplasm|mitochondrion
ILMN_1705774
NM_032862
TIGD5
84948
0
.0
0
0
3
0
7
1
0
.0
6
3
2
0
.0
0
0
2
1
6
4
5
1
.9
1
0
7
3
2
6
6
2
8
q24.3
DNA binding|molecular_function|biological_process|regulation of transcription, DNA-
dependent|cellular_component|chromosome, centromeric region|nucleus
ILMN_1723211
NM_024884
L2HGDH
79944
0
.0
0
0
3
9
7
1
0
.0
6
8
8
0
.0
0
0
2
1
6
4
5
1
.6
1
3
5
3
1
6
5
3
1
4
q21.3
2
-
hydroxyglutarate dehydrogenase activity|oxidoreductase activity|2-oxoglutarate metabolic
process|cellular protein metabolic process|small molecule metabolic process|integral to
membrane|integral to mitochondrial inner membrane|mitochondrial inner
membrane|mitochondrion
ILMN_1661940
NM_015215
CAMTA1
23261
0
.0
0
0
4
0
3
2
0
.0
6
8
8
0
.0
0
0
2
1
6
4
5
-
2
.2
5
1
5
3
8
7
6
7
1
p36.31-p36.23
DNA binding|regulation of transcription, DNA-dependent|cytoplasm|nucleus
ILMN_1711899
NM_001002857
ANXA2
302
0
.0
0
0
4
1
7
9
0
.0
6
9
0
.0
0
0
2
1
6
4
5
-
1
.7
8
5
8
7
5
1
9
5
1
5
q22.2
Rab GTPase binding|calcium ion binding|calcium-dependent phospholipid
binding|cytoskeletal protein binding|phosphatidylinositol-4,5-bisphosphate
binding|phospholipase inhibitor activity|protein binding|angiogenesis|body fluid
secretion|cellular response to acid|collagen fibril organization|fibrinolysis|positive
regulation of binding|positive regulation of vesicle fusion|skeletal system
development|Schmidt-Lanterman incisure|basement membrane|cytoplasm|early
endosome|extracellular matrix|extracellular region|extrinsic to plasma
membrane|melanosome|midbody|myelin sheath adaxonal region|perinuclear region of
cytoplasm|plasma membrane|protein complex|sarcolemma|soluble fraction                                                  


ILMN_1683450
NM_080668
CDCA5
113130
0
.0
0
0
4
2
8
0
.0
6
9
4
0
.0
0
0
2
1
6
4
5
1
.8
2
3
7
4
9
3
6
1
1
q12.1
chromatin binding|protein binding|G1/S transition of mitotic cell cycle|cell cycle|cell
division|double-strand break repair|interphase of mitotic cell cycle|mitosis|mitotic
chromosome condensation|mitotic metaphase plate congression|mitotic sister chromatid
cohesion|regulation of cohesin localization to chromatin|chromosome|cohesin
complex|cytoplasm|nuclear chromatin|nucleolus|nucleus|nucleus|plasma membrane
ILMN_1697189
NM_001039582
PNCK
139728
0
.0
0
0
4
6
2
2
0
.0
7
0
9
0
.0
0
0
2
1
6
4
5
2
.4
4
8
9
0
0
9
5
1
Xq28
ATP binding|calmodulin binding|calmodulin-dependent protein kinase activity|nucleotide
binding|cytoplasm|nucleus
ILMN_1777591
NM_138413
C10orf65
112817
0
.0
0
0
4
6
3
5
0
.0
7
0
9
0
.0
0
0
2
1
6
4
5
2
.3
2
4
8
1
0
6
0
3
1
0
q24.2
4
-
hydroxy-2-oxoglutarate aldolase activity|lyase activity|glyoxylate catabolic
process|mitochondrion
ILMN_1777397
NM_002448
MSX1
4487
0
.0
0
0
4
6
9
6
0
.0
7
0
9
0
.0
0
0
2
1
6
4
5
3
.1
1
4
3
6
7
0
2
5
4
p16.2
RNA polymerase II core promoter proximal region sequence-specific DNA binding
transcription factor activity|p53 binding|sequence-specific DNA binding|sequence-specific
DNA binding transcription factor activity|BMP signaling pathway involved in heart
development|anterior/posterior pattern specification|apoptotic nuclear change|bone
morphogenesis|cartilage morphogenesis|cell morphogenesis|embryonic forelimb
morphogenesis|embryonic hindlimb morphogenesis|embryonic nail plate
morphogenesis|epithelial to mesenchymal transition|epithelial to mesenchymal transition
involved in endocardial cushion formation|face morphogenesis|forebrain
development|heart morphogenesis|in utero embryonic development|mammary gland
epithelium development|mesenchymal cell proliferation|midbrain development|middle ear
morphogenesis|multicellular organismal development|muscle organ development|negative
regulation of apoptotic process|negative regulation of cell growth|negative regulation of cell
proliferation|negative regulation of striated muscle cell differentiation|negative regulation of
transcription from RNA polymerase II promoter|negative regulation of transcription
ILMN_1716704
NM_032206
NLRC5
84166
0
.0
0
0
4
8
6
6
0
.0
7
1
5
0
.0
0
0
2
1
6
4
5
-
2
.4
7
3
9
3
1
1
8
8
1
6
q13
ATP binding|RNA polymerase II core promoter sequence-specific DNA binding|nucleotide
binding|protein binding|defense response to virus|innate immune response|innate immune
response|negative regulation of NF-kappaB transcription factor activity|negative regulation
of type I interferon production|negative regulation of type I interferon-mediated signaling
pathway|positive regulation of MHC class I biosynthetic process|positive regulation of
interferon-gamma-mediated signaling pathway|positive regulation of interferon-gamma-
mediated signaling pathway|positive regulation of transcription from RNA polymerase II
promoter|positive regulation of type I interferon-mediated signaling pathway|regulation of
kinase activity|cytoplasm|cytosol|cytosol|nucleus
ILMN_1738684
NM_138734
NRXN2
9379
0
.0
0
0
5
3
7
4
0
.0
7
3
7
0
.0
0
0
2
1
6
4
5
2
.9
2
0
2
9
3
3
1
1
q13
calcium channel regulator activity|cell adhesion molecule binding|metal ion binding|cell
adhesion|gephyrin clustering|neuroligin clustering|neurotransmitter secretion|postsynaptic
density protein 95 clustering|postsynaptic membrane assembly|synaptic
transmission|integral to membrane|membrane
ILMN_2394362
NM_199138
FAM123A
219287
0
.0
0
0
5
7
7
6
0
.0
7
3
8
0
.0
0
0
2
1
6
4
5
3
.2
6
6
0
3
6
8
9
4
1
3
q12.13
phosphatidylinositol-4,5-bisphosphate binding|protein binding|ectoderm
development|negative regulation of canonical Wnt receptor signaling pathway|plasma
membrane
ILMN_1792571
NM_173728
ARHGEF15
22899
0
.0
0
0
6
2
3
8
0
.0
7
6
2
0
.0
0
0
2
1
6
4
5
-
2
.9
4
3
4
1
6
4
7
2
1
7
p13.1
GTPase activator activity|Rho guanyl-nucleotide exchange factor activity|Rho guanyl-
nucleotide exchange factor activity|guanyl-nucleotide exchange factor activity|protein
binding|negative regulation of synapse maturation|positive regulation of stress fiber
assembly|regulation of Rho protein signal transduction|regulation of catalytic activity|cell
projection|cytoplasm|dendrite|intracellular
ILMN_2407346
NM_194436
LDHD
197257
0
.0
0
0
6
7
2
6
0
.0
7
9
4
0
.0
0
0
2
1
6
4
5
1
.5
9
4
5
4
8
5
5
1
6
q23.1
D-lactate dehydrogenase (cytochrome) activity|UDP-N-acetylmuramate dehydrogenase
activity|flavin adenine dinucleotide binding|oxidoreductase activity, acting on CH-OH group
of donors|protein binding|mitochondrial inner membrane|mitochondrion                                          


ILMN_1777233
NM_004091
E2F2
1870
0
.0
0
0
7
0
2
4
0
.0
7
9
4
0
.0
0
0
2
1
6
4
5
1
.6
5
5
3
5
1
8
2
9
1
p36
DNA binding|core promoter binding|protein binding|sequence-specific DNA binding
transcription factor activity|transcription factor binding|G1 phase of mitotic cell
cycle|apoptotic process|mitotic cell cycle|regulation of cell cycle|regulation of transcription,
DNA-dependent|transcription initiation from RNA polymerase II
promoter|nucleoplasm|nucleus|transcription factor complex
ILMN_1745282
NM_014226
RAGE
5891
0
.0
0
0
7
1
2
2
0
.0
7
9
4
0
.0
0
0
2
1
6
4
5
0
.8
9
5
3
0
2
6
2
1
1
4
q32
ATP binding|cyclin-dependent protein kinase activity|nucleotide binding|protein kinase
activity|protein serine/threonine kinase activity|protein phosphorylation|signal
transduction|cytoplasm
ILMN_2073592
NM_012298
CAND2
23066
0
.0
0
0
7
1
6
1
0
.0
7
9
4
0
.0
0
0
2
1
6
4
5
1
.9
4
4
8
5
8
4
4
6
3
p25.2
TBP-class protein binding|protein binding|positive regulation of transcription, DNA-
dependent|intracellular|nucleus
ILMN_1778625
NM_000610
CD44
960
0
.0
0
0
7
2
3
1
0
.0
7
9
4
0
.0
0
0
4
3
2
9
-
2
.0
5
8
8
9
3
6
8
9
1
1
p13
binding|collagen binding|hyaluronic acid binding|hyaluronic acid binding|hyaluronic acid
binding|hyalurononglucosaminidase activity|protein binding|receptor
activity|transmembrane signaling receptor activity|cartilage development|cell adhesion|cell-
cell adhesion|cell-matrix adhesion|cytokine-mediated signaling pathway|hyaluronan
catabolic process|interferon-gamma-mediated signaling pathway|monocyte
aggregation|negative regulation of DNA damage response, signal transduction by p53 class
mediator|negative regulation of apoptotic process|negative regulation of apoptotic
process|negative regulation of cysteine-type endopeptidase activity involved in apoptotic
process|positive regulation of ERK1 and ERK2 cascade|positive regulation of heterotypic cell-
cell adhesion|positive regulation of peptidyl-serine phosphorylation|positive regulation of
peptidyl-tyrosine phosphorylation|cell surface|integral to plasma
membrane|membrane|plasma membrane
ILMN_2381730
NM_016215
EGFL7
51162
0
.0
0
0
7
6
5
9
0
.0
7
9
4
0
.0
0
0
2
1
6
4
5
-
2
.2
5
1
5
3
8
7
6
7
9
q34.3
calcium ion binding|angiogenesis|blood vessel development|multicellular organismal
development|negative regulation of cell migration|vasculogenesis|extracellular
region|extracellular space
ILMN_1718132
NM_004092
ECHS1
1892
0
.0
0
0
7
7
8
0
.0
7
9
4
0
.0
0
0
2
1
6
4
5
2
.1
6
0
2
7
4
8
3
1
1
0
q26.2-q26.3
enoyl-CoA hydratase activity|lyase activity|protein binding|cellular lipid metabolic
process|fatty acid beta-oxidation|fatty acid metabolic process|small molecule metabolic
process|mitochondrial matrix|mitochondrion|mitochondrion|soluble fraction
ILMN_3308183
NR_003233
SNORD113-5
767565
0
.0
0
0
7
9
1
0
.0
7
9
4
0
.0
0
0
8
6
5
8
-
2
.5
5
6
3
9
3
3
4
9
ILMN_2350574
NM_138373
MYADM
91663
0
.0
0
0
7
9
7
4
0
.0
7
9
4
0
.0
0
0
2
1
6
4
5
-
2
.1
2
0
2
9
4
2
3
4
1
9
q13.42
integral to membrane|membrane
ILMN_1656452
NM_025108
C16orf59
80178
0
.0
0
0
8
6
0
7
0
.0
7
9
4
0
.0
0
0
2
1
6
4
5
3
.5
5
9
4
9
1
8
1
3
1
6
p13.3
ILMN_1805665
NM_198391
FLRT3
23767
0
.0
0
0
8
6
9
8
0
.0
7
9
4
0
.0
0
0
4
3
2
9
2
.0
1
7
9
2
1
9
0
8
2
0
p11
protein binding, bridging|receptor signaling protein activity|biological_process|cell
adhesion|integral to membrane|integral to plasma membrane|membrane|proteinaceous
extracellular matrix
ILMN_1695414
NM_018154
ASF1B
55723
0
.0
0
0
8
8
1
8
0
.0
7
9
4
0
.0
0
0
2
1
6
4
5
1
.9
0
3
0
3
8
2
7
1
9
p13.12
histone binding|cell differentiation|chromatin assembly or disassembly|chromatin
modification|multicellular organismal development|nucleosome assembly|regulation of
transcription, DNA-dependent|spermatogenesis|chromatin|nucleus                                              


ILMN_2188264
NM_001554
CYR61
3491
0
.0
0
0
8
8
7
4
0
.0
7
9
4
0
.0
0
0
6
4
9
3
5
-
2
.1
2
0
2
9
4
2
3
4
1
p22.3
extracellular matrix binding|heparin binding|insulin-like growth factor binding|integrin
binding|anatomical structure morphogenesis|apoptosis involved in heart
morphogenesis|atrial septum morphogenesis|atrioventricular valve morphogenesis|cell
proliferation|chemotaxis|chondroblast differentiation|chorio-allantoic fusion|extracellular
matrix organization|intussusceptive angiogenesis|labyrinthine layer blood vessel
development|negative regulation of apoptotic process|positive regulation of BMP signaling
pathway|positive regulation of apoptotic process|positive regulation of cartilage
development|positive regulation of cell migration|positive regulation of cell-substrate
adhesion|positive regulation of ceramide biosynthetic process|positive regulation of cysteine-
type endopeptidase activity involved in apoptotic process|positive regulation of osteoblast
differentiation|positive regulation of osteoblast proliferation|positive regulation of
phospholipase activity|positive regulation of protein kinase activity|positive regulation of
protein phosphorylation|positive regulation of transcription from RNA polymerase II
promoter|reactive oxygen species metabolic process|regulation of ERK1 and ERK2
ILMN_1676348
NM_024689
CXorf36
79742
0
.0
0
0
9
0
3
8
0
.0
7
9
4
0
.0
0
0
2
1
6
4
5
-
2
.9
4
3
4
1
6
4
7
2
Xp11.3
extracellular region
ILMN_1788813
NM_033315
RASL10B
91608
0
.0
0
0
9
1
1
8
0
.0
7
9
4
0
.0
0
0
2
1
6
4
5
2
.0
5
3
1
1
1
3
3
6
1
7
q12
GTP binding|GTPase activity|nucleotide binding|signal transduction|small GTPase mediated
signal transduction|plasma membrane
ILMN_2098643
NM_021727
FADS3
3995
0
.0
0
0
9
3
1
4
0
.0
7
9
4
0
.0
0
0
2
1
6
4
5
-2
1
1
q12-q13.1
heme binding|molecular_function|oxidoreductase activity|oxidoreductase activity, acting on
paired donors, with oxidation of a pair of donors resulting in the reduction of molecular
oxygen to two molecules of water|electron transport chain|transport|unsaturated fatty acid
biosynthetic process|endoplasmic reticulum|endoplasmic reticulum membrane|integral to
membrane|membrane|membrane fraction
ILMN_2294762
NM_004038
AMY1A
276
0
.0
0
0
9
3
7
3
0
.0
7
9
4
0
.0
0
0
2
1
6
4
5
1
.6
5
0
7
6
4
5
5
9
1
p21
alpha-amylase activity|hydrolase activity, acting on glycosyl bonds|metal ion binding|protein
binding|carbohydrate metabolic process|digestion|extracellular region
ILMN_1690066
NM_145715
TIGD2
166815
0
.0
0
0
9
7
1
5
0
.0
8
1
5
0
.0
0
0
2
1
6
4
5
1
.8
7
5
7
8
0
0
6
3
4
q22.1
DNA binding|regulation of transcription, DNA-dependent|chromosome, centromeric
region|nucleus
ILMN_1779711
NM_016448
DTL
51514
0
.0
0
1
0
4
2
1
0
.0
8
3
5
0
.0
0
0
2
1
6
4
5
1
.9
4
1
1
0
6
3
1
1
1
q32
protein binding|ubiquitin-protein ligase activity|DNA replication|G2/M transition DNA
damage checkpoint|protein monoubiquitination|protein polyubiquitination|protein
polyubiquitination|regulation of cell cycle|response to DNA damage stimulus|response to
UV|translesion synthesis|ubiquitin-dependent protein catabolic process|ubiquitin-
dependent protein catabolic process|Cul4A-RING ubiquitin ligase complex|Cul4B-RING
ubiquitin ligase complex|centrosome|cytoplasm|cytoskeleton|membrane|nuclear
membrane|nucleus
ILMN_1654468
NM_020115
ACRV1
56
0
.0
0
1
0
5
8
6
0
.0
8
3
5
0
.0
0
0
2
1
6
4
5
2
.1
1
1
0
3
1
3
1
2
ILMN_1678968
NM_005261
GEM
2669
0
.0
0
1
0
8
5
6
0
.0
8
4
3
0
.0
0
0
4
3
2
9
-
1
.8
3
6
5
0
1
2
6
8
8
q13-q21
GDP binding|GTP binding|GTPase activity|calmodulin binding|magnesium ion
binding|nucleotide binding|protein binding|GTP catabolic process|cell surface receptor
signaling pathway|immune response|signal transduction|small GTPase mediated signal
transduction|internal side of plasma membrane|plasma membrane
ILMN_2320277
NM_020109
ACRV1
56
0
.0
0
1
1
2
2
4
0
.0
8
5
5
0
.0
0
0
2
1
6
4
5
2
.1
4
7
3
0
6
6
9
9
ILMN_3310321
NR_003231
SNORD113-3
767563
0
.0
0
1
1
2
7
3
0
.0
8
5
5
0
.0
0
0
2
1
6
4
5
-
2
.3
2
1
9
2
8
0
9
5
ILMN_2077952
NM_020692
GALNTL1
57452
0
.0
0
1
1
6
3
5
0
.0
8
6
6
0
.0
0
0
2
1
6
4
5
2
.0
1
4
3
5
5
2
9
3
1
4
q24.1
polypeptide N-acetylgalactosaminyltransferase activity|transferase activity, transferring
glycosyl groups|carbohydrate metabolic process|Golgi apparatus|Golgi membrane|integral
to membrane|membrane
ILMN_1651657
NM_025220
ADAM33
80332
0
.0
0
1
2
0
7
5
0
.0
8
7
4
0
.0
0
0
2
1
6
4
5
-
4
.0
5
8
8
9
3
6
8
9
2
0
p13
metal ion binding|metalloendopeptidase activity|metalloendopeptidase activity|peptidase
activity|zinc ion binding|zinc ion binding|integrin-mediated signaling
pathway|proteolysis|proteolysis|integral to membrane|membrane
ILMN_3308555
NR_003235
SNORD113-7
767567
0
.0
0
1
2
3
2
8
0
.0
8
9
5
0
.0
0
0
4
3
2
9
-
2
.3
9
5
9
2
8
6
7
6                                                    


ILMN_2117736
NM_013244
MGAT4C
25834
0
.0
0
1
2
6
5
7
0
.0
8
9
5
0
.0
0
0
4
3
2
9
2
.3
3
9
1
3
7
3
8
5
1
2
q21
alpha-1,3-mannosylglycoprotein 4-beta-N-acetylglucosaminyltransferase activity|metal ion
binding|transferase activity, transferring hexosyl groups|carbohydrate metabolic
process|cellular protein metabolic process|post-translational protein modification|protein N-
linked glycosylation via asparagine|Golgi apparatus|Golgi membrane|integral to
membrane|membrane
ILMN_2051381
NM_153329
ALDH16A1
126133
0
.0
0
1
4
4
6
4
0
.0
9
2
1
0
.0
0
0
2
1
6
4
5
-
2
.0
5
8
8
9
3
6
8
9
1
9
q13.33
oxidoreductase activity|oxidoreductase activity, acting on the aldehyde or oxo group of
donors, NAD or NADP as acceptor
ILMN_3241254
NR_002835
HAS2AS
594842
0
.0
0
1
5
2
9
5
0
.0
9
2
4
0
.0
0
0
2
1
6
4
5
2
.2
2
9
5
8
7
9
2
3
ILMN_2408851
NM_181720
ARHGAP30
257106
0
.0
0
1
5
9
5
9
0
.0
9
4
8
0
.0
0
0
2
1
6
4
5
-
2
.1
2
0
2
9
4
2
3
4
1
q23.3
GTPase activator activity|regulation of small GTPase mediated signal transduction|signal
transduction|small GTPase mediated signal transduction|cytoplasmic membrane-bounded
vesicle|cytosol|intracellular
ILMN_1755974
NM_005165
ALDOC
230
0
.0
0
1
6
6
2
1
0
.0
9
5
6
0
.0
0
0
2
1
6
4
5
2
.9
7
0
8
5
3
6
5
4
1
7
cen-q12
cytoskeletal protein binding|fructose-bisphosphate aldolase activity|lyase activity|protein
binding|aging|apoptotic process|carbohydrate metabolic process|fructose 1,6-bisphosphate
metabolic process|fructose metabolic process|gluconeogenesis|glucose metabolic
process|glycolysis|organ regeneration|protein heterotetramerization|protein
homotetramerization|response to hypoxia|response to organic cyclic compound|response to
organic nitrogen|small molecule metabolic
process|axon|cytoplasm|cytoskeleton|cytosol|mitochondrion
ILMN_1730546
NM_020988
GNAO1
2775
0
.0
0
1
7
0
1
8
0
.0
9
6
0
.0
0
0
8
6
5
8
2
.5
1
8
5
3
5
1
3
9
1
6
q13
G-protein beta/gamma-subunit complex binding|G-protein coupled serotonin receptor
binding|GTP binding|GTPase activity|guanyl nucleotide binding|metal ion binding|mu-type
opioid receptor binding|nucleotide binding|phosphatidylinositol phospholipase C
activity|signal transducer activity|G-protein signaling, coupled to cAMP nucleotide second
messenger|GTP catabolic process|GTP catabolic process|aging|cellular process|dopamine
receptor signaling pathway|forebrain development|locomotory behavior|muscle
contraction|negative regulation of calcium ion transport|neuron projection
development|positive regulation of GTPase activity|regulation of heart contraction|response
to cytokine stimulus|response to drug|response to hydrogen peroxide|response to
morphine|response to organic cyclic compound|response to organic nitrogen|extrinsic to
internal side of plasma membrane|heterotrimeric G-protein complex|intracellular|plasma
membrane|synaptosome
ILMN_3308753
NR_003193
SNORD114-1
767577
0
.0
0
1
8
0
4
5
0
.0
9
9
1
0
.0
0
0
2
1
6
4
5
-
2
.0
5
8
8
9
3
6
8
9
ILMN_2210386
NM_198276
TMEM17
200728
0
.0
0
1
8
9
8
2
0
.1
0
1
0
.0
0
0
4
3
2
9
2
.0
3
2
1
0
0
8
4
3
2
p15
cell projection organization|cilium assembly|smoothened signaling pathway|TCTN-B9D
complex|ciliary transition zone|cilium|cilium membrane|integral to membrane|plasma
membrane
ILMN_1728581
NM_016210
C3orf18
51161
0
.0
0
1
9
3
8
1
0
.1
0
2
0
.0
0
0
4
3
2
9
-
1
.7
8
5
8
7
5
1
9
5
3
p21.3
integral to membrane|membrane
ILMN_2227790
NM_000835
GRIN2C
2905
0
.0
0
1
9
5
6
4
0
.1
0
2
0
.0
0
0
4
3
2
9
2
.0
2
5
0
2
8
7
9
4
1
7
q25
N-methyl-D-aspartate selective glutamate receptor activity|cation channel
activity|extracellular-glutamate-gated ion channel activity|ion channel activity|ionotropic
glutamate receptor activity|receptor activity|transporter activity|cation transport|directional
locomotion|glutamate receptor signaling pathway|ion transmembrane transport|ion
transport|negative regulation of protein catabolic process|neuromuscular process controlling
balance|protein localization|regulation of excitatory postsynaptic membrane
potential|regulation of membrane potential|response to wounding|signal
transduction|synaptic transmission|transport|N-methyl-D-aspartate selective glutamate
receptor complex|cell junction|cytoplasm|integral to membrane|integral to plasma
membrane|outer membrane-bounded periplasmic space|plasma membrane|plasma
membrane|postsynaptic density|postsynaptic membrane|synapse                                            


ILMN_1659792
NM_014213
HOXD9
3235
0
.0
0
1
9
6
0
9
0
.1
0
2
0
.0
0
0
2
1
6
4
5
-
2
.1
8
4
4
2
4
5
7
1
2
q31.1
sequence-specific DNA binding|sequence-specific DNA binding transcription factor
activity|adult locomotory behavior|anterior/posterior pattern specification|embryonic
forelimb morphogenesis|embryonic skeletal system morphogenesis|hindlimb
morphogenesis|mammary gland development|multicellular organismal
development|peripheral nervous system neuron development|positive regulation of
transcription from RNA polymerase II promoter|proximal/distal pattern formation|skeletal
muscle tissue development|nucleus
ILMN_1787280
NM_024037
C1orf135
79000
0
.0
0
1
9
6
5
8
0
.1
0
2
0
.0
0
0
6
4
9
3
5
2
.4
0
0
5
3
7
9
3
1
p36.11
ILMN_1663772
NM_016932
SIX2
10736
0
.0
0
1
9
8
4
6
0
.1
0
2
0
.0
0
0
2
1
6
4
5
-
1
.7
8
5
8
7
5
1
9
5
2
p21
protein complex binding|sequence-specific DNA binding|sequence-specific DNA binding
transcription factor activity|anatomical structure morphogenesis|cell
proliferation|chondrocyte differentiation|embryonic cranial skeleton morphogenesis|kidney
development|mesenchymal to epithelial transition involved in metanephros
morphogenesis|mesodermal cell fate specification|metanephros development|middle ear
morphogenesis|multicellular organismal development|negative regulation of cell
differentiation|positive regulation of transcription from RNA polymerase II promoter|protein
import into nucleus|regulation of transcription, DNA-dependent|nucleus
ILMN_2201596
NM_018659
CYTL1
54360
0
.0
0
2
1
1
5
0
.1
0
6
0
.0
0
0
6
4
9
3
5
2
.3
7
2
9
5
2
0
9
8
4
p16-p15
receptor binding|chondrocyte differentiation|chondroitin sulfate proteoglycan biosynthetic
process|positive regulation of sequence-specific DNA binding transcription factor
activity|positive regulation of transcription from RNA polymerase II promoter|signal
transduction|extracellular region|extracellular space|soluble fraction
ILMN_1701006
NM_144608
HEXIM2
124790
0
.0
0
2
1
7
5
9
0
.1
0
8
0
.0
0
0
4
3
2
9
2
.0
7
0
3
8
9
3
2
8
1
7
q21.31
cyclin-dependent protein kinase inhibitor activity|protein binding|snRNA binding|negative
regulation of cyclin-dependent protein kinase activity|negative regulation of transcription
from RNA polymerase II promoter|negative regulation of transcription, DNA-
dependent|cytoplasm|cytoplasm|nucleolus|nucleus|nucleus
ILMN_1695978
NM_152570
LINGO2
158038
0
.0
0
2
2
2
5
3
0
.1
0
8
0
.0
0
0
4
3
2
9
4
.3
2
9
8
4
1
1
7
7
9
p21.2
integral to membrane|membrane
ILMN_1755954
NM_014912
CPEB3
22849
0
.0
0
2
4
1
3
1
0
.1
1
2
0
.0
0
0
2
1
6
4
5
2
.4
3
5
6
2
8
5
9
4
1
0
q23.32
RNA binding|nucleotide binding
ILMN_2225061
NM_001039111
TRIM71
131405
0
.0
0
2
5
5
4
2
0
.1
1
2
0
.0
0
0
4
3
2
9
2
.1
3
7
5
0
3
5
2
4
ILMN_1698038
NM_032222
FAM188B
84182
0
.0
0
2
6
8
7
6
0
.1
1
4
0
.0
0
0
6
4
9
3
5
2
.0
9
4
2
3
6
0
7
7
p14.3
ILMN_2400326
NM_003582
DYRK3
8444
0
.0
0
2
7
2
9
9
0
.1
1
4
0
.0
0
0
2
1
6
4
5
-
2
.3
2
1
9
2
8
0
9
5
1
q32.1
ATP binding|ATP binding|magnesium ion binding|nucleotide binding|protein kinase
activity|protein kinase activity|protein serine/threonine kinase activity|protein
serine/threonine/tyrosine kinase activity|protein tyrosine kinase activity|erythrocyte
differentiation|protein phosphorylation|nucleus
ILMN_2068257
NM_032348
MXRA8
54587
0
.0
0
2
7
5
6
5
0
.1
1
4
0
.0
0
0
2
1
6
4
5
-
2
.0
5
8
8
9
3
6
8
9
1
p36.33
integral to membrane|membrane
ILMN_2311089
NM_007304
BRCA1
672
0
.0
0
2
7
8
9
2
0
.1
1
4
0
.0
0
0
4
3
2
9
2
.0
4
6
1
4
1
7
8
2
ILMN_1793002
NM_152504
C20orf196
149840
0
.0
0
2
8
1
3
3
0
.1
1
5
0
.0
0
0
4
3
2
9
2
.6
5
5
3
5
1
8
2
9
2
0
p12.3
ILMN_1805395
NM_021070
LTBP3
4054
0
.0
0
2
8
4
0
4
0
.1
1
5
0
.0
0
0
2
1
6
4
5
-
3
.0
5
8
8
9
3
6
8
9
1
1
q13.1
binding|calcium ion binding|growth factor binding|bone morphogenesis|bone
remodeling|lung saccule development|negative regulation of bone mineralization|negative
regulation of chondrocyte differentiation|positive regulation of bone resorption|skeletal
system development|transforming growth factor beta receptor signaling
pathway|extracellular region                                                    


ILMN_1670540
NM_005900
SMAD1
4086
0
.0
0
2
8
5
6
8
0
.1
1
5
0
.0
0
0
4
3
2
9
-
2
.0
5
8
8
9
3
6
8
9
4
q31
I-
SMAD binding|RNA polymerase II core promoter sequence-specific DNA binding|co-SMAD
binding|identical protein binding|protein binding|protein kinase binding|receptor signaling
protein activity|sequence-specific DNA binding transcription factor activity|transforming
growth factor beta receptor, pathway-specific cytoplasmic mediator activity|BMP signaling
pathway|BMP signaling pathway|BMP signaling pathway|MAPK cascade|SMAD protein
complex assembly|cellular response to organic cyclic compound|embryonic pattern
specification|gamete generation|hindbrain development|homeostatic process|inflammatory
response|kidney development|mesodermal cell fate commitment|midbrain
development|negative regulation of cell proliferation|osteoblast fate commitment|positive
regulation of anti-apoptosis|positive regulation of cartilage development|positive regulation
of dendrite morphogenesis|positive regulation of gene expression|positive regulation of
osteoblast differentiation|positive regulation of transcription from RNA polymerase II
promoter|primary miRNA processing|protein phosphorylation|regulation of transcription,
DNA-dependent|response to drug|response to organic nitrogen|signal
ILMN_3310451
NR_003224
SNORD114-31
767612
0
.0
0
2
8
5
7
1
0
.1
1
5
0
.0
0
0
4
8
7
0
1
3
-
2
.0
5
8
8
9
3
6
8
9
ILMN_3310416
NR_003195
SNORD114-3
767579
0
.0
0
2
9
6
4
7
0
.1
1
6
0
.0
0
0
2
1
6
4
5
-
2
.3
2
1
9
2
8
0
9
5
ILMN_3251587
NR_003287
LOC100008589 100008589
0
.0
0
3
0
.1
1
6
0
.0
0
0
4
3
2
9
-
3
.6
6
2
0
0
3
5
3
6
ILMN_1799062
NM_001077493
NFKB2
4791
0
.0
0
3
0
3
2
9
0
.1
1
6
0
.0
0
0
2
1
6
4
5
-
1
.8
3
6
5
0
1
2
6
8
1
0
q24
DNA binding|protein binding|sequence-specific DNA binding transcription factor
activity|transcription coactivator activity|MyD88-dependent toll-like receptor signaling
pathway|MyD88-independent toll-like receptor signaling pathway|TRIF-dependent toll-like
receptor signaling pathway|Toll signaling pathway|extracellular matrix organization|follicular
dendritic cell differentiation|germinal center formation|innate immune response|positive
regulation of NF-kappaB transcription factor activity|regulation of transcription, DNA-
dependent|signal transduction|spleen development|toll-like receptor 1 signaling
pathway|toll-like receptor 2 signaling pathway|toll-like receptor 3 signaling pathway|toll-like
receptor 4 signaling pathway|toll-like receptor signaling pathway|Bcl3/NF-kappaB2
complex|cytoplasm|cytosol|nucleolus|nucleoplasm|nucleus
ILMN_2359029
NM_020642
C11orf17
56672
0
.0
0
3
2
9
0
.1
1
9
0
.0
0
0
2
1
6
4
5
1
.9
4
8
6
0
0
8
4
7
1
1
p15.3
protein binding|substrate adhesion-dependent cell spreading|nucleus
ILMN_2356632
NM_003581
NCK2
8440
0
.0
0
3
3
0
2
9
0
.1
1
9
0
.0
0
0
2
1
6
4
5
-
2
.1
8
4
4
2
4
5
7
1
2
q12
cytoskeletal adaptor activity|protein binding|receptor signaling complex scaffold activity|T
cell activation|actin filament organization|axon guidance|cell migration|epidermal growth
factor receptor signaling pathway|lamellipodium assembly|negative regulation of cell
proliferation|positive regulation of T cell proliferation|positive regulation of actin filament
polymerization|positive regulation of transcription from RNA polymerase II
promoter|regulation of epidermal growth factor-activated receptor activity|regulation of
translation|signal complex assembly|signal transduction|cytoplasm|cytosol|endoplasmic
reticulum|vesicle membrane
ILMN_1750674
NM_138432
SDSL
113675
0
.0
0
3
4
2
2
0
.1
2
0
.0
0
0
4
3
2
9
2
.0
1
4
3
5
5
2
9
3
1
2
q24.13
L-
serine ammonia-lyase activity|L-threonine ammonia-lyase activity|lyase
activity|molecular_function|pyridoxal phosphate binding|biological_process|cellular amino
acid metabolic process|cellular_component|mitochondrion
ILMN_3236736
NM_001136273
ZFP92
139735
0
.0
0
3
7
7
0
7
0
.1
2
3
0
.0
0
0
4
3
2
9
-
2
.0
5
8
8
9
3
6
8
9
Xq28
DNA binding|metal ion binding|zinc ion binding|regulation of transcription, DNA-
dependent|intracellular|nucleus                                          


ILMN_1792710
NM_001348
DAPK3
1613
0
.0
0
3
7
7
2
5
0
.1
2
3
0
.0
0
0
4
3
2
9
-2
1
9
p13.3
ATP binding|leucine zipper domain binding|nucleotide binding|protein homodimerization
activity|protein serine/threonine kinase activity|protein serine/threonine kinase
activity|chromatin modification|cytokinesis|induction of apoptosis|induction of
apoptosis|intracellular protein kinase cascade|neuron differentiation|positive regulation of
canonical Wnt receptor signaling pathway|protein autophosphorylation|protein
autophosphorylation|protein phosphorylation|regulation of actin cytoskeleton
reorganization|regulation of apoptotic process|regulation of autophagy|regulation of cell
motility|regulation of mitosis|regulation of smooth muscle contraction|regulation of
transcription, DNA-dependent|PML body|chromosome|chromosome, centromeric
region|cytoplasm|cytoskeleton|microtubule organizing center|nucleus
ILMN_3236808
NR_024149
MEG8
79104
0
.0
0
3
8
3
7
8
0
.1
2
4
0
.0
0
0
6
4
9
3
5
-2
ILMN_1682763
NM_000477
ALB
213
0
.0
0
3
9
3
8
7
0
.1
2
4
0
.0
0
0
4
3
2
9
-
2
.2
5
1
5
3
8
7
6
7
4
q13.3
DNA binding|antioxidant activity|cell surface binding|chaperone binding|copper ion
binding|drug binding|drug binding|enzyme binding|fatty acid binding|fatty acid
binding|metal ion binding|oxygen binding|protein binding|pyridoxal phosphate
binding|toxin binding|zinc ion binding|bile acid and bile salt transport|bile acid metabolic
process|blood coagulation|cellular response to starvation|hemolysis by symbiont of host
erythrocytes|lipid metabolic process|lipoprotein metabolic process|maintenance of
mitochondrion location|negative regulation of apoptotic process|negative regulation of
programmed cell death|platelet activation|platelet degranulation|positive regulation of
circadian sleep/wake cycle, non-REM sleep|response to mercury ion|response to
nutrient|response to organic substance|response to platinum ion|response to stress|small
molecule metabolic process|sodium-independent organic anion transport|transmembrane
transport|transport|transport|basement membrane|cytoplasm|extracellular
region|extracellular region|extracellular space|extracellular space|platelet alpha granule
lumen|protein complex
ILMN_1701906
NM_006678
CD300C
10871
0
.0
0
3
9
7
6
2
0
.1
2
4
0
.0
0
0
6
4
9
3
5
2
.0
4
9
6
3
0
7
6
8
1
7
q25.1
receptor activity|transmembrane signaling receptor activity|cellular defense
response|integral to plasma membrane|plasma membrane
ILMN_1751666
NM_015597
GPSM1
26086
0
.0
0
4
0
0
2
2
0
.1
2
4
0
.0
0
0
6
4
9
3
5
-
2
.2
5
1
5
3
8
7
6
7
9
q34.3
G-protein alpha-subunit binding|GDP-dissociation inhibitor activity|GTPase regulator
activity|binding|cell differentiation|multicellular organismal development|nervous system
development|regulation of G-protein coupled receptor protein signaling pathway|signal
transduction|Golgi apparatus|Golgi membrane|cytoplasm|cytosol|endoplasmic
reticulum|endoplasmic reticulum membrane|nucleolus|nucleus|plasma membrane
ILMN_1669523
NM_005252
FOS
2353
0
.0
0
4
0
2
5
5
0
.1
2
4
0
.0
0
0
8
6
5
8
-
2
.3
9
5
9
2
8
6
7
6
1
4
q24.3
R-SMAD binding|double-stranded DNA binding|protein binding|protein dimerization
activity|sequence-specific DNA binding|sequence-specific DNA binding transcription factor
activity|transcription regulatory region DNA binding|DNA methylation|MyD88-dependent
toll-like receptor signaling pathway|MyD88-independent toll-like receptor signaling
pathway|SMAD protein signal transduction|TRIF-dependent toll-like receptor signaling
pathway|Toll signaling pathway|aging|cellular response to calcium ion|cellular response to
extracellular stimulus|cellular response to hormone stimulus|cellular response to reactive
oxygen species|conditioned taste aversion|female pregnancy|inflammatory response|innate
immune response|nervous system development|positive regulation of transcription from
RNA polymerase II promoter|positive regulation of transcription, DNA-dependent|regulation
of sequence-specific DNA binding transcription factor activity|regulation of transcription from
RNA polymerase II promoter|response to cAMP|response to cold|response to corticosterone
stimulus|response to cytokine stimulus|response to drug|response to gravity|response to
light stimulus|response to lipopolysaccharide|response to mechanical stimulus|response to
ILMN_1806752
NM_172069
PLEKHH2
130271
0
.0
0
4
1
8
9
6
0
.1
2
5
0
.0
0
0
4
3
2
9
-2
2
p21
binding|cytoplasm|cytoskeleton|integral to membrane|membrane                                      


ILMN_1698996
NM_194255
SLC19A1
6573
0
.0
0
4
4
1
0
3
0
.1
2
7
0
.0
0
0
6
4
9
3
5
-
2
.1
2
0
2
9
4
2
3
4
2
1
q22.3
folic acid binding|folic acid transporter activity|methotrexate transporter activity|reduced
folate carrier activity|folic acid metabolic process|folic acid transport|methotrexate
transport|small molecule metabolic process|transport|vitamin metabolic process|water-
soluble vitamin metabolic process|integral to plasma membrane|membrane fraction|plasma
membrane
ILMN_1697670
NM_005839
SRRM1
10250
0
.0
0
4
5
1
0
2
0
.1
2
9
0
.0
0
0
8
6
5
8
2
.0
6
3
5
0
2
9
4
2
1
p36.11
DNA binding|RNA binding|RNA splicing|RNA splicing, via transesterification reactions|gene
expression|mRNA 3'-end processing|mRNA export from nucleus|nuclear mRNA splicing, via
spliceosome|nuclear mRNA splicing, via spliceosome|termination of RNA polymerase II
transcription|transcription from RNA polymerase II promoter|catalytic step 2
spliceosome|cytosol|nuclear matrix|nuclear speck|nucleoplasm|nucleus
ILMN_3234837
NM_138370
PKDCC
91461
0
.0
0
4
7
3
7
1
0
.1
3
2
0
.0
0
0
4
3
2
9
-
2
.1
8
4
4
2
4
5
7
1
2
p21
ATP binding|nucleotide binding|protein kinase activity|cell differentiation|embryonic
digestive tract development|lung alveolus development|multicellular organismal
development|negative regulation of Golgi to plasma membrane protein
transport|ossification|palate development|positive regulation of bone
mineralization|positive regulation of chondrocyte differentiation|protein
phosphorylation|protein transport|Golgi apparatus
ILMN_1733045
NM_004914
RAB36
9609
0
.0
0
4
9
6
0
6
0
.1
3
4
0
.0
0
0
6
4
9
3
5
2
.6
3
2
2
6
8
2
1
5
2
2
q11.22
GTP binding|nucleotide binding|protein transport|small GTPase mediated signal
transduction|Golgi apparatus|Golgi membrane|membrane
ILMN_3243037
NR_024015
TDRG1
732253
0
.0
0
4
9
6
8
0
.1
3
4
0
.0
0
0
8
6
5
8
2
.3
7
2
9
5
2
0
9
8
ILMN_1697081
NM_033036
GAL3ST3
89792
0
.0
0
5
0
7
7
1
0
.1
3
4
0
.0
0
0
6
4
9
3
5
2
.4
3
5
6
2
8
5
9
4
1
1
q13.1
3
'-
phosphoadenosine 5'-phosphosulfate binding|carbohydrate binding|galactose 3-O-
sulfotransferase activity|galactose 3-O-sulfotransferase activity|galactosylceramide
sulfotransferase activity|proteoglycan sulfotransferase activity|transferase
activity|biosynthetic process|monosaccharide metabolic process|oligosaccharide metabolic
process|poly-N-acetyllactosamine metabolic process|proteoglycan biosynthetic
process|sulfur compound metabolic process|Golgi apparatus|Golgi cisterna
membrane|integral to membrane|membrane
ILMN_1748352
NM_001333
CTSL2
1515
0
.0
0
5
1
4
2
8
0
.1
3
4
0
.0
0
0
2
7
0
5
6
3
2
.0
3
2
1
0
0
8
4
3
9
q22.2
aminopeptidase activity|cysteine-type endopeptidase activity|cysteine-type peptidase
activity|kininogen binding|peptidase activity|peptide binding|Sertoli cell
differentiation|autophagic cell death|cell communication|cellular response to
starvation|decidualization|multicellular organismal aging|nerve
development|proteolysis|response to glucocorticoid stimulus|response to glucose
stimulus|response to gonadotropin stimulus|response to organic cyclic
compound|spermatogenesis|apical part of cell|cytoplasm|external side of plasma
membrane|lysosome|microvillus|neuron projection|perikaryon|secretory granule|soluble
fraction|vacuole
ILMN_1712023
NM_182936
SLC8A3
6547
0
.0
0
5
3
3
2
4
0
.1
3
4
0
.0
0
0
4
3
2
9
2
.8
4
5
9
9
1
7
7
1
1
4
q24.1
antiporter activity|calcium:sodium antiporter activity|calmodulin binding|blood
coagulation|calcium ion transport|calcium ion transport into cytosol|cell
communication|cellular response to cAMP|ion transport|sodium ion
transport|telencephalon development|transmembrane transport|transmembrane
transport|cell projection|dendritic spine|integral to
membrane|membrane|microtubule|mitochondrion|neuronal cell body|plasma
membrane|sarcolemma
ILMN_1687084
NM_173654
C3orf64
285203
0
.0
0
5
3
6
3
4
0
.1
3
4
0
.0
0
0
2
1
6
4
5
-
1
.8
3
6
5
0
1
2
6
8
3
p14.1
transferase activity, transferring glycosyl groups|extracellular region
ILMN_1778845
NM_022074
FAM111A
63901
0
.0
0
5
3
6
5
5
0
.1
3
4
0
.0
0
0
4
3
2
9
-
1
.8
3
6
5
0
1
2
6
8
1
1
q12.1
catalytic activity
ILMN_1773413
NM_015296
DOCK9
23348
0
.0
0
5
3
8
8
3
0
.1
3
4
0
.0
0
0
2
1
6
4
5
-
2
.1
8
4
4
2
4
5
7
1
1
3
q32.3
GTP binding|GTPase binding|guanyl-nucleotide exchange factor activity|protein
binding|biological_process|blood coagulation|cellular_component|cytosol|endomembrane
system|membrane                                              


ILMN_1695847
NM_145102
ZKSCAN5
23660
0
.0
0
5
7
4
1
4
0
.1
3
8
0
.0
0
0
6
4
9
3
5
2
.1
1
4
3
6
7
0
2
5
7
q22
DNA binding|metal ion binding|sequence-specific DNA binding transcription factor
activity|zinc ion binding|regulation of transcription, DNA-dependent|viral
reproduction|intracellular|nucleus
ILMN_2065745
NM_005856
RAMP3
10268
0
.0
0
5
7
8
6
8
0
.1
3
8
0
.0
0
0
8
6
5
8
-
2
.3
2
1
9
2
8
0
9
5
7
p13-p12
coreceptor activity|protein binding|protein transporter activity|receptor activity|calcium ion
transport|intracellular protein transport|negative regulation of transcription, DNA-
dependent|positive regulation of receptor recycling|protein localization in plasma
membrane|protein transport|receptor internalization|regulation of G-protein coupled
receptor protein signaling pathway|signal transduction|cell surface|integral to plasma
membrane|lysosome|plasma membrane|plasma membrane|plasma membrane|receptor
complex
ILMN_1795507
NM_080284
ABCA6
23460
0
.0
0
5
8
6
7
2
0
.1
3
8
0
.0
0
0
4
3
2
9
-
2
.1
8
4
4
2
4
5
7
1
1
7
q24.3
ATP binding|ATPase activity|nucleotide binding|transport|integral to membrane|membrane
ILMN_1768282
NM_001042632
SNX21
90203
0
.0
0
5
8
7
4
5
0
.1
3
8
0
.0
0
0
2
1
6
4
5
-
2
.5
5
6
3
9
3
3
4
9
2
0
q13.12
binding|lipid binding|phosphatidylinositol binding|cell communication|protein
transport|cytoplasmic vesicle|cytoplasmic vesicle membrane|membrane
ILMN_1712506
NM_130797
DPP6
1804
0
.0
0
5
9
4
8
4
0
.1
3
8
0
.0
0
0
6
4
9
3
5
2
.9
2
5
9
9
9
4
1
9
7
q36.2
dipeptidyl-peptidase activity|serine-type peptidase activity|cell death|proteolysis|integral to
membrane|membrane
ILMN_1715508
NM_006169
NNMT
4837
0
.0
0
6
0
4
0
7
0
.1
3
9
0
.0
0
0
2
1
6
4
5
-
2
.3
2
1
9
2
8
0
9
5
1
1
q23.1
methyltransferase activity|nicotinamide N-methyltransferase activity|transferase
activity|methylation|small molecule metabolic process|xenobiotic metabolic
process|cytoplasm|cytosol
ILMN_1769031
NM_004958
FRAP1
2475
0
.0
0
6
1
7
9
7
0
.1
4
0
.0
0
0
4
3
2
9
-
2
.0
5
8
8
9
3
6
8
9
1
p36.2
ATP binding|RNA polymerase III type 1 promoter DNA binding|RNA polymerase III type 2
promoter DNA binding|RNA polymerase III type 3 promoter DNA binding|TFIIIC-class
transcription factor binding|kinase activity|kinase activity|nucleotide
binding|phosphoprotein binding|phosphotransferase activity, alcohol group as
acceptor|protein binding|protein domain specific binding|protein serine/threonine kinase
activity|protein serine/threonine kinase activity|ribosome binding|T cell costimulation|TOR
signaling cascade|cell growth|cell growth|cellular response to hypoxia|cellular response to
nutrient levels|epidermal growth factor receptor signaling pathway|fibroblast growth factor
receptor signaling pathway|germ cell development|growth|insulin receptor signaling
pathway|negative regulation of NFAT protein import into nucleus|negative regulation of
autophagy|negative regulation of cell size|negative regulation of macroautophagy|nerve
growth factor receptor signaling pathway|peptidyl-serine phosphorylation|peptidyl-
threonine phosphorylation|phosphatidylinositol-mediated
signaling|phosphorylation|phosphorylation|positive regulation of actin filament
ILMN_2230025
NM_014476
PDLIM3
27295
0
.0
0
6
2
8
0
2
0
.1
4
2
0
.0
0
0
8
6
5
8
-
2
.1
8
4
4
2
4
5
7
1
4
q35
actinin binding|metal ion binding|structural constituent of muscle|zinc ion binding|actin
filament organization|heart development|Z disc|actin cytoskeleton|cytoplasm
ILMN_1660635
NM_016027
LACTB2
51110
0
.0
0
6
5
4
0
8
0
.1
4
4
0
.0
0
0
6
4
9
3
5
2
.2
3
8
7
8
6
8
6
8
p22-q22.3
hydrolase activity|metal ion binding
ILMN_1754076
NM_018398
CACNA2D3
55799
0
.0
0
6
6
5
4
1
0
.1
4
4
0
.0
0
0
6
4
9
3
5
1
.8
8
3
6
2
0
8
1
6
3
p21.1
metal ion binding|voltage-gated calcium channel activity|voltage-gated ion channel
activity|ion transport|integral to membrane|membrane
ILMN_1652826
NM_005824
LRRC17
10234
0
.0
0
6
6
9
0
.1
4
4
0
.0
0
0
6
4
9
3
5
2
.2
8
9
8
3
4
4
6
5
7
q22.1
bone marrow development|negative regulation of osteoclast differentiation|osteoblast
differentiation|osteoblast proliferation|extracellular region|extracellular space
ILMN_1702125
NM_004502
HOXB7
3217
0
.0
0
6
7
8
5
2
0
.1
4
5
0
.0
0
0
6
4
9
3
5
2
.5
5
0
9
0
0
6
6
5
1
7
q21.3
protein binding|sequence-specific DNA binding|sequence-specific DNA binding transcription
factor activity|anterior/posterior pattern specification|embryonic skeletal system
morphogenesis|multicellular organismal development|myeloid cell differentiation|positive
regulation of branching involved in ureteric bud morphogenesis|regulation of transcription,
DNA-dependent|nucleus
ILMN_1662419
NM_001864
COX7A1
1346
0
.0
0
6
9
1
5
1
0
.1
4
6
0
.0
0
0
2
1
6
4
5
-
3
.1
8
4
4
2
4
5
7
1
1
9
q13.1
cytochrome-c oxidase activity|electron carrier activity|generation of precursor metabolites
and energy|integral to membrane|membrane|mitochondrial respiratory
chain|mitochondrion                                                  


ILMN_1740234
NM_183239
GSTO2
119391
0
.0
0
6
9
3
0
5
0
.1
4
7
0
.0
0
0
2
1
6
4
5
2
.2
5
3
9
8
9
2
6
6
1
0
q25.1
glutathione dehydrogenase (ascorbate) activity|glutathione transferase activity|glutathione
transferase activity|methylarsonate reductase activity|oxidoreductase activity|transferase
activity|L-ascorbic acid metabolic process|L-ascorbic acid metabolic process|cellular
response to arsenic-containing substance|metabolic process|oxidation-reduction
process|small molecule metabolic process|vitamin metabolic process|water-soluble vitamin
metabolic process|xenobiotic metabolic process|xenobiotic metabolic
process|cytoplasm|cytosol
ILMN_2125346
NM_024690
MUC16
94025
0
.0
0
7
0
3
1
3
0
.1
4
7
0
.0
0
0
3
2
4
6
7
5
2
1
9
p13.2
protein binding|O-glycan processing|cell adhesion|cellular protein metabolic process|post-
translational protein modification|Golgi lumen|extracellular region|extracellular
space|extrinsic to membrane|integral to membrane|plasma membrane
ILMN_1751530
NM_052850
GADD45GIP1
90480
0
.0
0
7
0
3
4
4
0
.1
4
7
0
.0
0
0
2
7
0
5
6
3
2
.0
4
2
6
4
4
3
3
7
1
9
p13.2
protein binding|cell cycle|interspecies interaction between
organisms|mitochondrion|nucleus
ILMN_2410713
NM_213647
FGFR4
2264
0
.0
0
7
5
4
6
0
.1
5
2
0
.0
0
0
4
3
2
9
2
.3
8
1
2
8
3
3
7
3
5
q35.1-qter
ATP binding|fibroblast growth factor binding|fibroblast growth factor-activated receptor
activity|fibroblast growth factor-activated receptor activity|heparin binding|nucleotide
binding|protein tyrosine kinase activity|receptor activity|cell migration|fibroblast growth
factor receptor signaling pathway|fibroblast growth factor receptor signaling
pathway|fibroblast growth factor receptor signaling pathway|fibroblast growth factor
receptor signaling pathway|glucose homeostasis|insulin receptor signaling pathway|peptidyl-
tyrosine phosphorylation|phosphate ion homeostasis|positive regulation of DNA biosynthetic
process|positive regulation of ERK1 and ERK2 cascade|positive regulation of cell
proliferation|positive regulation of cell proliferation|positive regulation of metalloenzyme
activity|positive regulation of proteolysis|protein autophosphorylation|regulation of bile acid
biosynthetic process|regulation of cholesterol homeostasis|regulation of extracellular matrix
disassembly|regulation of lipid metabolic process|cell-cell junction|cytoplasm|endoplasmic
reticulum|endosome|extracellular region|integral to plasma
membrane|nucleolus|nucleus|plasma membrane
ILMN_1731044
NM_023930
KCTD14
65987
0
.0
0
7
8
9
3
5
0
.1
5
3
0
.0
0
0
6
4
9
3
5
2
.4
4
3
6
0
6
6
5
1
1
1
q14.1
voltage-gated potassium channel activity|potassium ion transport|membrane|voltage-gated
potassium channel complex
ILMN_1742677
NM_006361
HOXB13
10481
0
.0
0
8
1
7
3
3
0
.1
5
6
0
.0
0
0
3
2
4
6
7
5
2
.3
0
1
5
8
7
6
4
7
1
7
q21.2
sequence-specific DNA binding|sequence-specific DNA binding transcription factor
activity|angiogenesis|epidermis development|epithelial cell maturation involved in prostate
gland development|morphogenesis of an epithelium|multicellular organismal
development|prostate epithelial cord arborization involved in prostate glandular acinus
morphogenesis|regulation of growth|response to testosterone stimulus|response to
wounding|nucleus|transcription factor complex
ILMN_1784203
NM_032489
ACRBP
84519
0
.0
0
8
5
1
5
9
0
.1
5
9
0
.0
0
0
2
1
6
4
5
2
.7
3
5
5
2
2
1
7
7
1
2
p13.31
molecular_function|biological_process|acrosomal vesicle|cellular_component|cytoplasmic
vesicle|extracellular region
ILMN_2111739
NM_006715
MAN2C1
4123
0
.0
0
8
7
8
2
9
0
.1
6
0
.0
0
0
6
4
9
3
5
-
2
.1
2
0
2
9
4
2
3
4
1
5
q11-q13
alpha-mannosidase activity|carbohydrate binding|metal ion binding|zinc ion
binding|carbohydrate metabolic process|mannose metabolic process
ILMN_1663754
NM_030824
ZNF442
79973
0
.0
0
9
2
4
6
6
0
.1
6
3
0
.0
0
0
2
7
0
5
6
3
2
.6
0
4
0
7
1
3
2
4
1
9
p13.2
DNA binding|metal ion binding|zinc ion binding|regulation of transcription, DNA-
dependent|intracellular|nucleus
ILMN_1744068
NM_018091
ELP3
55140
0
.0
0
9
2
6
2
9
0
.1
6
3
0
.0
0
0
3
2
4
6
7
5
2
.1
0
0
9
7
7
6
4
8
8
p21.1
DNA binding|histone acetyltransferase activity|iron-sulfur cluster binding|metal ion
binding|phosphorylase kinase regulator activity|protein binding|transferase activity,
transferring acyl groups|regulation of transcription from RNA polymerase II
promoter|regulation of transcription from RNA polymerase II promoter|transcription
elongation from RNA polymerase II promoter|DNA-directed RNA polymerase II,
holoenzyme|cytoplasm|nucleolus|nucleus|transcription elongation factor complex
ILMN_1746670
NM_173629
C18orf26
284254
0
.0
0
9
3
4
4
1
0
.1
6
3
0
.0
0
0
4
3
2
9
2
.2
0
7
8
9
2
8
5
2
1
8
q21.2
integral to membrane|membrane                                              


ILMN_2137789
NM_004235
KLF4
9314
0
.0
0
9
5
2
1
7
0
.1
6
5
0
.0
0
0
3
2
4
6
7
5
-
2
.2
5
1
5
3
8
7
6
7
9
q31
DNA binding|RNA polymerase II core promoter proximal region sequence-specific DNA
binding transcription factor activity involved in positive regulation of transcription|RNA
polymerase II transcription factor binding|RNA polymerase II transcription factor
binding|RNA polymerase II transcription factor binding|RNA polymerase II transcription
factor binding transcription factor activity involved in positive regulation of transcription|core
promoter proximal region sequence-specific DNA binding|double-stranded DNA
binding|metal ion binding|phosphatidylinositol 3-kinase regulator activity|sequence-specific
DNA binding|sequence-specific DNA binding transcription factor activity|sequence-specific
DNA binding transcription factor recruiting transcription factor activity|sequence-specific
DNA binding transcription factor recruiting transcription factor activity|transcription
regulatory region DNA binding|transcription regulatory region DNA binding|zinc ion
binding|cellular response to growth factor stimulus|epidermal cell differentiation|epidermis
morphogenesis|fat cell differentiation|fat cell differentiation|mesodermal cell fate
ILMN_1710571
NM_001040285
PAPD5
64282
0
.0
0
9
6
5
5
2
0
.1
6
5
0
.0
0
0
8
6
5
8
2
.0
0
3
6
0
2
2
3
7
1
6
q12.1
DNA binding|DNA-directed DNA polymerase activity|metal ion
binding|molecular_function|nucleotidyltransferase activity|transferase activity|DNA
replication|cell cycle|cell division|histone mRNA catabolic
process|mitosis|cellular_component|cytoplasm|nucleus
ILMN_1770892
NM_003403
YY1
7528
0
.0
0
9
9
9
4
2
0
.1
6
7
0
.0
0
0
2
7
0
5
6
3
0
.9
5
6
0
5
6
6
5
2
14q
DNA binding|RNA binding|four-way junction DNA binding|metal ion binding|protein
binding|sequence-specific DNA binding transcription factor activity|transcription coactivator
activity|transcription corepressor activity|transcription regulatory region DNA binding|zinc
ion binding|DNA recombination|DNA repair|RNA localization|anterior/posterior pattern
specification|camera-type eye morphogenesis|cell differentiation|cellular response to
UV|chromosome organization|double-strand break repair via homologous
recombination|negative regulation of transcription from RNA polymerase II
promoter|regulation of transcription from RNA polymerase II promoter|response to DNA
damage stimulus|response to UV-C|spermatogenesis|Ino80 complex|PcG protein
complex|intracellular|nuclear matrix|nucleus|plasma membrane|transcription factor
complex
ILMN_1651692
NM_005990
STK10
6793
0
.0
1
0
1
5
4
3
0
.1
6
8
0
.0
0
0
2
1
6
4
5
-
2
.1
2
0
2
9
4
2
3
4
5
q35.1
ATP binding|nucleotide binding|polo kinase kinase activity|protein binding|protein
homodimerization activity|protein serine/threonine kinase activity|cell cycle|lymphocyte
aggregation|protein autophosphorylation|protein phosphorylation|regulation of lymphocyte
migration|plasma membrane
ILMN_2329114
NM_080539
COLQ
8292
0
.0
1
0
2
4
0
7
0
.1
6
8
0
.0
0
0
4
3
2
9
-
2
.1
2
0
2
9
4
2
3
4
3
p25
heparin binding|protein binding|acetylcholine catabolic process in synaptic cleft|asymmetric
protein localization|neurotransmitter catabolic process|basal lamina|cell
junction|collagen|extracellular space|synapse|synaptic cleft
ILMN_1773168
NM_201564
SYCE1
93426
0
.0
1
0
3
7
1
0
.1
6
9
0
.0
0
0
4
3
2
9
-
3
.3
6
5
8
7
1
4
4
2
ILMN_2393765
NM_020070
IGLL1
3543
0
.0
1
0
7
9
9
8
0
.1
7
0
.0
0
0
2
7
0
5
6
3
-
1
.8
8
8
9
6
8
6
8
8
2
2
q11.23
immune response|extracellular region|membrane
ILMN_1794588
NM_003583
DYRK2
8445
0
.0
1
1
0
7
8
9
0
.1
7
2
0
.0
0
0
2
1
6
4
5
-
2
.1
8
4
4
2
4
5
7
1
1
2
q15
ATP binding|magnesium ion binding|manganese ion binding|nucleotide binding|protein
kinase activity|protein serine/threonine kinase activity|protein serine/threonine/tyrosine
kinase activity|protein tyrosine kinase activity|ubiquitin binding|DNA damage response,
signal transduction by p53 class mediator resulting in induction of apoptosis|apoptotic
process|positive regulation of glycogen biosynthetic process|protein
phosphorylation|response to DNA damage stimulus|smoothened signaling
pathway|cytoplasm|intracellular membrane-bounded
organelle|nucleolus|nucleus|ubiquitin ligase complex
ILMN_1671149
NR_002766
MEG3
55384
0
.0
1
1
1
2
8
4
0
.1
7
2
0
.0
0
0
3
7
8
7
8
8
-
2
.3
2
1
9
2
8
0
9
5
ILMN_1701551
NM_080284
ABCA6
23460
0
.0
1
1
4
6
0
2
0
.1
7
3
0
.0
0
0
4
3
2
9
-
2
.6
4
3
8
5
6
1
9
1
7
q24.3
ATP binding|ATPase activity|nucleotide binding|transport|integral to membrane|membrane                                            


ILMN_2148785
NM_002053
GBP1
2633
0
.0
1
1
8
2
9
1
0
.1
7
4
0
.0
0
0
4
3
2
9
-
2
.4
7
3
9
3
1
1
8
8
1
p22.2
GTP binding|GTPase activity|nucleotide binding|cytokine-mediated signaling
pathway|interferon-gamma-mediated signaling pathway|cytosol|plasma membrane
ILMN_1803560
NM_022040
LAT2
7462
0
.0
1
2
1
1
1
1
0
.1
7
4
0
.0
0
0
4
8
7
0
1
3
2
.5
8
4
9
6
2
5
0
1
ILMN_2076600
NM_004867
ITM2A
9452
0
.0
1
2
4
1
1
7
0
.1
7
7
0
.0
0
0
4
3
2
9
-
2
.7
3
6
9
6
5
5
9
4
Xq13.3-Xq21.2
protein binding|integral to membrane|membrane
ILMN_1672608
NM_003713
PPAP2B
8613
0
.0
1
2
4
2
8
1
0
.1
7
7
0
.0
0
0
4
3
2
9
-
2
.3
2
1
9
2
8
0
9
5
1
p32.2
hydrolase activity|integrin binding|lipid phosphatase activity|phosphatidate phosphatase
activity|phosphoprotein phosphatase activity|protein binding|blood vessel
development|canonical Wnt receptor signaling pathway involved in positive regulation of cell-
cell adhesion|canonical Wnt receptor signaling pathway involved in positive regulation of
endothelial cell migration|canonical Wnt receptor signaling pathway involved in positive
regulation of wound healing|dephosphorylation|gastrulation with mouth forming
second|germ cell migration|homotypic cell-cell adhesion|lipid metabolic process|negative
regulation of protein phosphorylation|phospholipid metabolic process|positive regulation of
peptidyl-tyrosine phosphorylation|positive regulation of sequence-specific DNA binding
transcription factor activity|protein stabilization|regulation of Wnt receptor signaling
pathway|small molecule metabolic process|sphingolipid biosynthetic process|sphingolipid
metabolic process|Golgi apparatus|adherens junction|endoplasmic reticulum
membrane|integral to membrane|membrane|plasma membrane
ILMN_2108938
NM_015308
FNBP4
23360
0
.0
1
2
6
1
7
5
0
.1
7
8
0
.0
0
0
2
1
6
4
5
-
2
.0
5
8
8
9
3
6
8
9
1
1
p11.2
ILMN_2407703
NM_006950
SYN1
6853
0
.0
1
2
7
2
4
4
0
.1
7
9
0
.0
0
0
3
7
8
7
8
8
2
.3
1
6
1
4
5
7
4
2
Xp11.23
ATP binding|actin binding|ligase activity|transporter activity|neurotransmitter
secretion|synaptic transmission|transport|Golgi apparatus|cell junction|intracellular
organelle|presynaptic active zone|soluble fraction|synapse|synaptic vesicle
membrane|synaptonemal complex
ILMN_2297864
NM_001077525
MTMR14
64419
0
.0
1
3
6
8
7
4
0
.1
8
3
0
.0
0
0
6
4
9
3
5
-2
3
p26
hydrolase activity|phosphatase activity|phosphatidylinositol-3-phosphatase activity|protein
tyrosine phosphatase activity|cytoplasm|perinuclear region of cytoplasm|ruffle
ILMN_1672547
NM_004145
MYO9B
4650
0
.0
1
3
9
7
2
8
0
.1
8
4
0
.0
0
0
2
1
6
4
5
-
2
.1
8
4
4
2
4
5
7
1
1
9
p13.1
ADP binding|ATP binding|ATPase activity|GTPase activator activity|Rho GTPase activator
activity|actin binding|calmodulin binding|metal ion binding|microfilament motor
activity|nucleotide binding|protein binding|protein homodimerization activity|ARF protein
signal transduction|ATP catabolic process|Rho protein signal transduction|actin filament-
based movement|positive regulation of Rho GTPase activity|regulation of small GTPase
mediated signal transduction|small GTPase mediated signal transduction|actin
cytoskeleton|cell cortex|cytoplasm|cytosol|filamentous actin|myosin complex|perinuclear
region of cytoplasm
ILMN_1737170
NM_002018
FLII
2314
0
.0
1
4
2
3
2
7
0
.1
8
6
0
.0
0
0
6
4
9
3
5
-
2
.1
8
4
4
2
4
5
7
1
1
7
p11.2
actin binding|protein binding|multicellular organismal development|muscle
contraction|regulation of transcription, DNA-
dependent|centrosome|cytoplasm|cytoskeleton|nucleolus|nucleus                                          


ILMN_1713749
NM_007074
CORO1A
11151
0
.0
1
4
4
1
2
6
0
.1
8
6
0
.0
0
0
6
4
9
3
5
-
2
.0
5
8
8
9
3
6
8
9
1
6
p11.2
actin filament binding|cytoskeletal protein binding|phosphatidylinositol 3-kinase
binding|protein C-terminus binding|protein binding|protein homodimerization activity|T cell
homeostasis|actin cytoskeleton organization|actin filament organization|calcium ion
transport|cell-substrate adhesion|cellular component movement|homeostasis of number of
cells within a tissue|innate immune response|leukocyte chemotaxis|negative regulation of
actin nucleation|phagocytosis|phagolysosome assembly|positive chemotaxis|positive
regulation of T cell proliferation|positive regulation of cell migration|regulation of actin
filament polymerization|regulation of cell shape|response to cytokine stimulus|uropod
organization|actin filament|cell cortex|cortical actin cytoskeleton|cytoplasm|cytoplasmic
vesicle|immunological synapse|lamellipodium|nucleus|phagocytic cup|phagocytic
vesicle|phagocytic vesicle membrane|plasma membrane|protein complex
ILMN_2405667
NM_138429
CLDN15
24146
0
.0
1
5
1
6
3
2
0
.1
9
0
.0
0
0
8
6
5
8
-2
ILMN_1734827
NM_002417
MKI67
4288
0
.0
1
5
1
8
7
5
0
.1
9
0
.0
0
0
8
6
5
8
2
.2
2
0
3
2
9
9
5
5
1
0
q26.2
ATP binding|nucleotide binding|protein C-terminus binding|protein binding|DNA metabolic
process|cell cycle|cell proliferation|cellular response to heat|meiosis|organ
regeneration|chromosome, centromeric region|condensed
chromosome|cytoplasm|intracellular|nucleolus|nucleus
ILMN_3311085
NR_003194
SNORD114-2
767578
0
.0
1
5
1
9
4
5
0
.1
9
0
.0
0
0
5
4
1
1
2
5
-
2
.3
9
5
9
2
8
6
7
6
ILMN_1736567
NM_001025159
CD74
972
0
.0
1
5
2
1
0
.1
9
0
.0
0
0
8
6
5
8
-
0
.8
6
2
4
9
6
4
7
6
5
q32
MHC class II protein binding|MHC class II protein binding|beta-amyloid binding|cytokine
binding|cytokine receptor activity|identical protein binding|nitric-oxide synthase binding|T
cell selection|activation of MAPK activity|antigen processing and presentation of endogenous
antigen|antigen processing and presentation of exogenous peptide antigen via MHC class
II|cell proliferation|chaperone mediated protein folding requiring cofactor|cytokine-
mediated signaling pathway|defense response|immune response|immunoglobulin mediated
immune response|intracellular protein transport|macrophage migration inhibitory factor
signaling pathway|negative regulation of DNA damage response, signal transduction by p53
class mediator|negative regulation of T cell differentiation|negative regulation of apoptotic
process|negative regulation of apoptotic process|negative regulation of mature B cell
apoptosis|negative regulation of peptide secretion|negative thymic T cell selection|positive
regulation of B cell proliferation|positive regulation of ERK1 and ERK2 cascade|positive
regulation of ERK1 and ERK2 cascade|positive regulation of T cell differentiation|positive
regulation of chemokine (C-X-C motif) ligand 2 production|positive regulation of cytokine-
ILMN_2412571
NM_020461
TUBGCP6
85378
0
.0
1
5
3
4
8
2
0
.1
9
1
0
.0
0
0
4
3
2
9
-
2
.3
9
5
9
2
8
6
7
6
2
2
q13.31-q13.33
microtubule binding|G2/M transition of mitotic cell cycle|microtubule nucleation|mitotic cell
cycle|centrosome|cytoplasm|cytoskeleton|cytosol|gamma-tubulin ring
complex|microtubule|spindle pole
ILMN_1776640
NM_005373
MPL
4352
0
.0
1
5
4
1
4
2
0
.1
9
1
0
.0
0
0
4
8
7
0
1
3
0
.8
5
5
9
8
9
6
9
7
1
p34
cytokine receptor activity|receptor activity|transmembrane signaling receptor activity|blood
coagulation|cell proliferation|cell surface receptor signaling pathway|platelet
activation|integral to plasma membrane|membrane|plasma membrane|plasma membrane
ILMN_2338038
NM_013410
AK3L1
205
0
.0
1
5
5
1
9
7
0
.1
9
1
0
.0
0
0
3
2
4
6
7
5
0
.8
5
5
9
8
9
6
9
7
1
p31.3
ATP binding|GTP binding|adenylate kinase activity|nucleotide binding|transferase
activity|nucleobase-containing compound metabolic process|mitochondrial
matrix|mitochondrion
ILMN_1700811
NM_019116
UBFD1
56061
0
.0
1
5
5
9
4
0
.1
9
1
0
.0
0
0
5
4
1
1
2
5
-
0
.9
1
5
9
3
5
7
3
5
1
6
p12
ILMN_1685275
NM_006500
MCAM
4162
0
.0
1
5
6
0
1
9
0
.1
9
1
0
.0
0
0
5
4
1
1
2
5
-
0
.8
6
2
4
9
6
4
7
6
1
1
q23.3
anatomical structure morphogenesis|cell adhesion|glomerular filtration|vascular wound
healing|external side of plasma membrane|integral to membrane|plasma membrane                                            


ILMN_1724609
NM_014580
SLC2A8
29988
0
.0
1
5
9
0
9
7
0
.1
9
2
0
.0
0
0
3
7
8
7
8
8
0
.8
7
9
7
0
5
7
6
6
9
q33.3
glucose binding|glucose transmembrane transporter activity|substrate-specific
transmembrane transporter activity|transmembrane transporter activity|carbohydrate
metabolic process|carbohydrate transport|glucose transport|hexose transmembrane
transport|insulin receptor signaling pathway|male meiosis I|response to
hypoxia|transmembrane transport|cytoplasmic vesicle|cytoplasmic vesicle
membrane|integral to membrane|integral to plasma membrane|intracellular membrane-
bounded organelle|membrane|plasma membrane|synaptic vesicle
ILMN_2122420
NM_000191
HMGCL
3155
0
.0
1
5
9
3
1
4
0
.1
9
2
0
.0
0
0
6
4
9
3
5
2
1
p36.1-p35
carboxylic acid binding|fatty-acyl-CoA binding|hydroxymethylglutaryl-CoA lyase
activity|hydroxymethylglutaryl-CoA lyase activity|lyase activity|magnesium ion
binding|manganese ion binding|metal ion binding|protein homodimerization activity|acyl-
CoA metabolic process|cellular ketone body metabolic process|cellular lipid metabolic
process|embryo development|ketone body biosynthetic process|ketone body biosynthetic
process|leucine catabolic process|liver development|mitochondrion organization|response
to fatty acid|response to nutrient|response to starvation|small molecule metabolic
process|mitochondrial inner membrane|mitochondrial matrix|mitochondrial
matrix|mitochondrion|peroxisome
ILMN_3235718
NR_024448
LOC91316
91316
0
.0
1
5
9
4
8
2
0
.1
9
2
0
.0
0
0
4
8
7
0
1
3
-
0
.9
1
5
9
3
5
7
3
5
ILMN_1717975
NM_032687
CYHR1
50626
0
.0
1
5
9
8
4
6
0
.1
9
2
0
.0
0
0
2
7
0
5
6
3
-
2
.1
2
0
2
9
4
2
3
4
8
q24.3
metal ion binding|zinc ion binding|cytoplasm|nuclear envelope|perinuclear region of
cytoplasm
ILMN_1756928
NM_021136
RTN1
6252
0
.0
1
6
0
0
1
7
0
.1
9
2
0
.0
0
0
3
2
4
6
7
5
0
.9
0
3
0
3
8
2
7
1
4
q23.1
signal transducer activity|neuron differentiation|signal transduction|endoplasmic
reticulum|endoplasmic reticulum membrane|integral to endoplasmic reticulum
membrane|integral to membrane|membrane
ILMN_2229922
NM_018169
C12orf35
55196
0
.0
1
6
1
4
9
9
0
.1
9
3
0
.0
0
0
2
1
6
4
5
-2
1
2
p11.21
ILMN_2067708
NM_022366
TFB2M
64216
0
.0
1
6
2
0
0
7
0
.1
9
3
0
.0
0
0
2
7
0
5
6
3
-
0
.8
8
8
9
6
8
6
8
8
1
q44
methyltransferase activity|rRNA (adenine-N6,N6-)-dimethyltransferase activity|rRNA
methyltransferase activity|transcription cofactor activity|transferase activity|gene
expression|positive regulation of transcription, DNA-dependent|transcription from
mitochondrial promoter|transcription from mitochondrial promoter|transcription initiation
from mitochondrial promoter|transcription initiation from mitochondrial
promoter|mitochondrial matrix|mitochondrial matrix|mitochondrial nucleoid
ILMN_1760314
NM_032040
CCDC8
83987
0
.0
1
6
4
4
5
4
0
.1
9
5
0
.0
0
0
3
2
4
6
7
5
-
0
.8
8
8
9
6
8
6
8
8
1
9
q13.32
negative regulation of phosphatase activity|plasma membrane
ILMN_1722445
NM_005912
MC4R
4160
0
.0
1
6
6
4
7
3
0
.1
9
5
0
.0
0
0
3
7
8
7
8
8
2
.4
7
7
6
7
7
3
2
8
1
8
q22
G-protein coupled receptor activity|melanocortin receptor activity|melanocyte-stimulating
hormone receptor activity|neuropeptide binding|peptide hormone binding|protein
binding|receptor activity|signal transducer activity|ubiquitin protein ligase binding|G-protein
signaling, coupled to cAMP nucleotide second messenger|diet induced
thermogenesis|energy reserve metabolic process|feeding behavior|insulin secretion|positive
regulation of bone resorption|positive regulation of cAMP biosynthetic process|regulation of
metabolic process|response to insulin stimulus|integral to membrane|plasma membrane
ILMN_1813836
NM_001349
DARS
1615
0
.0
1
6
7
7
6
8
0
.1
9
6
0
.0
0
0
3
7
8
7
8
8
0
.8
5
5
9
8
9
6
9
7
2
q21.3
ATP binding|aminoacylase activity|aspartate-tRNA ligase activity|ligase activity|nucleic acid
binding|nucleotide binding|protein binding|aspartyl-tRNA aminoacylation|gene
expression|protein complex assembly|tRNA aminoacylation for protein
translation|translation|cytoplasm|cytosol|soluble fraction                                            


ILMN_1724234
NM_080706
TRPV1
7442
0
.0
1
6
7
7
7
8
0
.1
9
6
0
.0
0
0
3
2
4
6
7
5
-
0
.8
6
2
4
9
6
4
7
6
1
7
p13.2
ATP binding|calcium channel activity|calmodulin binding|chloride channel regulator
activity|nucleotide binding|behavioral response to pain|calcium ion transmembrane
transport|catagen|cell surface receptor signaling pathway|cellular response to growth factor
stimulus|cellular response to temperature stimulus|chemosensory behavior|detection of
chemical stimulus involved in sensory perception of pain|diet induced
thermogenesis|elevation of cytosolic calcium ion concentration|fever generation|glutamate
secretion|lipid metabolic process|microglial cell activation|negative regulation of
establishment of blood-brain barrier|peptide secretion|positive regulation of apoptotic
process|positive regulation of gastric acid secretion|positive regulation of nitric oxide
biosynthetic process|response to heat|response to pH|response to peptide hormone
stimulus|sensory perception of mechanical stimulus|smooth muscle contraction involved in
micturition|temperature homeostasis|thermoception|transport|cell
junction|cytosol|dendrite|dendritic spine membrane|integral to membrane|integral to
plasma membrane|neuronal cell body|plasma membrane|postsynaptic membrane|synapse
ILMN_1662161
NM_018201
TBC1D13
54662
0
.0
1
6
8
4
9
6
0
.1
9
7
0
.0
0
0
5
4
1
1
2
5
0
.8
8
7
5
2
5
2
7
1
9
q34.11
GTPase activator activity|Rab GTPase activator activity|intracellular
ILMN_2232368
NM_022104
PCIF1
63935
0
.0
1
6
8
7
5
3
0
.1
9
7
0
.0
0
0
8
6
5
8
-2
2
0
q13.12
negative regulation of phosphatase activity|nucleus                              
