# Supplementary material for: Identification of a gene expression driven progression pathway in myxoid liposarcoma
Source: Oncotarget. 2014 May 27;5(15):5965–77. doi: 10.18632/oncotarget.2023 (PMC4171605; doi:10.18632/oncotarget.2023)
Supplement: Supplementary file 7 [file oncotarget-05-5965-s007.doc]

| **Gene** | **Functions and regulating mechanisms** |
| --- | --- |
| **HOX gene family** | The HOX gene family to which *HOXB7* and *MSX1* belong are transcription factors mainly involved in tissue remodelling. They may act as oncogenes or tumour suppression genes depending on the context, but they are considered to be tumour modulators rather than onco-suppressors. Although mainly involved in controlling the proliferation of endothelial, vascular muscle, and multipotent cells [22], they play pleiotrophic roles sustained by means of various mechanisms [23,24]. The most frequent mechanism of HOX deregulation is epigenetic deregulation. HOX genes may be modulated by polycomb (PcG) and trithorax (TrxG) group protein complexes that lead to epigenetic changes by introducing the active H3K4m3 histone or repressive H3K27m3 modification to the HOX loci, which respectively lead to the activation or silencing of HOX genes. Other mechanisms of deregulation are mediated by non-coding RNAs [25], PARP, and protein-protein interactions. Finally, HOX genes may function as the hub of multiple growth factor pathways affecting the expression of crucial growth factors and enhancing the paracrine effects of the stromal component [23, 26]. |
| **YY1** | The *YYI* gene is a transcription factor that plays an essential role in embryonic development, and its aberrant expression is involved in various cancers. It is called Yin Yang (YY1) because it acts in seemingly ways depending on the stimuli received and the recruited co-factors, which are mainly represented by proteins that modulate histones and DNA. The activating co-factors include p300/CBP and protein arginine methyltransferase (PRMT)4, and the repressive co-factors include HDACs and Ezh2. Upon oncogenic stimulation, YY1 activates multiple oncogenes, including c-MYC [27]. At mRNA level, YY1 may lead to c-Myc over-expression as a result of: i) its own over-expression; ii) PRMT1-mediated methylation at Arg3 of histone H4 (H4-R3); or iii) disruption of the p300/HDAC3/YY1 complex [28]. However, it has recently been demonstrated that YY1 has DNA binding or transcription-independent activities, and that its functions may also be mediated by protein regulating modifiers interacting with YY1 at post-transcriptional level. These include acetylation and deacetylation via p300 and HDAC2, and methylation and demethylation via PRMT1 and Ezh2 [27,29]. |
| **c-MYC *(gene expression not modulated, but increased at protein level)*** | c-MYC is a pleiotropic factor mainly involved in cell cycle regulation and cell proliferation. It has recently been demonstrated that c-MYC does not regulate a unique set of target genes, but functions as an amplifier of the expression of already “ON” genes by increasing their transcription at all active promoters and enhancers [30,31]. These genes include YY1, which may be an active component of the c-MYC transcription network [32]. Together with OCT4, SOX2 and KLF4, c-MYC is also one of the OSKM factors whose ectopic expression induces the conversion of mesenchymal fibroblasts to pluripotency, thus driving the reprogramming of ESCs/iPSCs [33]; however, more recent insights suggest that reprogramming may also be driven by the direct transfection of miRNAs [34]. Currently, the ESC transcriptome programme recognises three distinct modules: core, polycomb and MYC. The MYC module is mainly activated by cell cycle structures to favour a fast cell cycle with a short G1/S phase ratio [35] in ES, iPS and piPS [36], as well as in the corresponding ESC-like cancer cells [37]. |

**Supplementary Table S6**
